# A network biology workflow to study transcriptomics data of the diabetic liver

**DOI:** 10.1186/1471-2164-15-971

**Published:** 2014-11-15

**Authors:** Martina Kutmon, Chris T Evelo, Susan L Coort

**Affiliations:** Department of Bioinformatics - BiGCaT, NUTRIM School for Nutrition, Toxicology and Metabolism, Maastricht University Maastricht, Kragujevac, The Netherlands; Maastricht Centre for Systems Biology (MaCSBio), Maastricht University, Maastricht, The Netherlands

**Keywords:** Transcriptomics, Network biology, Pathway analysis, Type 2 diabetes mellitus, Fatty liver, Non-alcoholic fatty liver disease, WikiPathways, PathVisio, Cytoscape

## Abstract

**Background:**

Nowadays a broad collection of transcriptomics data is publicly available in online repositories. Methods for analyzing these data often aim at deciphering the influence of gene expression at the process level. Biological pathway diagrams depict known processes and capture the interactions of gene products and metabolites, information that is essential for the computational analysis and interpretation of transcriptomics data.

The present study describes a comprehensive network biology workflow that integrates differential gene expression in the human diabetic liver with pathway information by building a network of interconnected pathways. Worldwide, the incidence of *type 2 diabetes mellitus* is increasing dramatically, and to better understand this multifactorial disease, more insight into the concerted action of the disease-related processes is needed. The liver is a key player in metabolic diseases and diabetic patients often develop non-alcoholic fatty liver disease.

**Results:**

A publicly available dataset comparing the liver transcriptome from lean and healthy vs. obese and insulin-resistant subjects was selected after a thorough analysis. Pathway analysis revealed seven significantly altered pathways in the WikiPathways human pathway collection. These pathways were then merged into one combined network with 408 gene products, 38 metabolites and 5 pathway nodes. Further analysis highlighted 17 nodes present in multiple pathways, and revealed the connections between different pathways in the network. The integration of transcription factor-gene interactions from the ENCODE project identified new links between the pathways on a regulatory level. The extension of the network with known drug-target interactions from DrugBank allows for a more complete study of drug actions and helps with the identification of other drugs that target proteins up- or downstream which might interfere with the action or efficiency of a drug.

**Conclusions:**

The described network biology workflow uses state-of-the-art pathway and network analysis methods to study the rewiring of the diabetic liver. The integration of experimental data and knowledge on disease-affected biological pathways, including regulatory elements like transcription factors or drugs, leads to improved insights and a clearer illustration of the overall process. It also provides a resource for building new hypotheses for further follow-up studies. The approach is highly generic and can be applied in different research fields.

**Electronic supplementary material:**

The online version of this article (doi:10.1186/1471-2164-15-971) contains supplementary material, which is available to authorized users.

## Background

*Type 2 diabetes mellitus* (T2DM) is a metabolic disorder characterized by chronic hyperglycemia with disturbances of carbohydrate, lipid and protein metabolism resulting from defects in insulin secretion, insulin resistance, or both. Obesity, the excess accumulation of lipids in the body, is a major risk factor for T2DM. Metabolism in the liver, adipose tissue and skeletal muscle is of key importance for the pathogenesis of T2DM. The current study focuses on the liver. It is well known that lipid accumulation in the liver contributes to insulin resistance, hyperglycemia and hyperlipidemia [1]. Hepatic lipid accumulation is the main characteristic of non-alcoholic fatty liver disease (NAFLD), and NAFLD is strongly associated with T2DM. In T2DM, one of the key liver functions, the postprandial insulin-mediated uptake of glucose, is impaired [2]. Moreover, gluconeogenesis is affected because of the disturbed insulin inhibition of glucose production [3]. Published studies of gene expression in liver of patients with NAFLD indicate an increase in both de novo lipogenesis and lipid oxidation [4, 5]. Although NAFLD studies have identified genes, proteins and processes that are important, not all biological mechanisms involved in the human diabetic liver have been deciphered [6].

Modern technology enables a global analysis of gene expression in liver tissue. Exploring published transcriptomics datasets available in online repositories revealed only one transcriptomics study investigating the human diabetic fatty liver. Pihlajamäki *et al*. [7] measured gene expression with microarray technology in liver biopsies of lean, obese and obese, diabetic subjects. Instead of only investigating the individual gene expression, current analysis methods often aim at deciphering biological functions at the process level. Generally, gene set enrichment analysis and pathway analysis are applied to find biological processes of interest [8]. Pihlajamäki *et al*. identified a relationship between thyroid hormone action and the altered gene expression pattern. The present study uses state-of-the-art pathway and network analysis methods to integrate knowledge of differential gene expression with pathway information by building a network of interconnected pathways. The focus shifts from looking at single gene expression levels to changes on process level and beyond.

Pathway analysis was used to find significantly altered biological processes. WikiPathways [9] was the database used to provide the pathway collections for this analysis. Next, network analysis was applied to i) identify the genes that link the pathways relevant in the diabetic fatty liver, ii) investigate the transcriptional regulation within the pathways and iii) identify known drugs that target genes in the pathways and their effects. The open-source and widely-used pathway and network visualization and analysis tools PathVisio [10] and Cytoscape [11] were used in a comprehensive network biology approach.

Although biological pathways are usually seen as independent processes, they do interact with and depend on each other. Our workflow combines and integrates relevant pathways into one biological network which allows researchers to study the effects of a disease or treatment not only in the pathway of interest but also in downstream or related pathways. Further extending the network with additional information, like transcription factor (TF) regulation, provides a more complete picture of complex biological processes and will promote the understanding of the mechanisms of diseases.

## Methods

### Transcriptomics dataset

In this study a published and publicly available transcriptomics dataset generated by Pihlajamäki [7] was used. The dataset is available from the Gene Expression Omnibus (accession number GSE15653). This was the only well-described human transcriptomics dataset on the human diabetic liver we found in online transcriptomics data repositories like GEO. 18 individuals, 5 lean and 13 obese, undergoing elective surgery for obesity or gallstones participated in the study. Based on a preoperative oral glucose tolerance test the obese subjects were diagnosed for T2DM. The percentage of liver fat content was significantly (p <0.05) increased in obese, diabetic subjects compared to lean subjects indicating the development of NAFLD in these subjects. Gene expression was measured in surgical liver biopsies from 4 obese subjects, 9 obese subjects with T2DM and 5 lean control subjects during fasting using Affymetrix Human Genome U133A microarrays. We selected the 5 lean and 9 obese, diabetic subjects to study the molecular changes in the diabetic fatty liver. The HOMA-IR (homeostatic model assessment for insulin resistance) shows a significantly higher insulin resistance in obese, diabetic subjects compared to lean subjects (p <0.05).

### Affymetrix microarray analysis

The raw data for 9 obese, diabetic subjects and 5 lean control subjects was reanalyzed with ArrayAnalysis.org, an online microarray quality control and pre-processing pipeline [12]. The data was normalized using the GC-RMA method and further evaluated. The quality control report is provided as Additional file 1.

The ArrayAnalysis.org statistics module uses the Limma package [13] of R/Bioconductor which applies linear regression models to make the statistical comparison between obese, diabetic subjects vs. control lean group. Genes were considered to be differentially expressed when their (1) absolute log2 fold change (FC) > 1 and (2) p-value < 0.05.

### Gene ontology analysis

Gene Ontology (GO) analysis was performed using the GO-Elite web-interface [14] to identify biological processes for the differentially expressed genes in the dataset. The following settings were used: (1) 2000 permutations, (2) Z-score pruning algorithm, (3) Z-score threshold > 1.96, (4) p-value threshold < 0.05 and (5) minimum number of changed genes is 3. GO-Elite uses an advanced ontology pruning algorithm to report a minimally non-redundant set of results.

### Pathway analysis

Pathway analysis was performed in PathVisio 3.1.3 (http://www.pathvisio.org) to interpret and visualize the molecular changes on a pathway level. The human pathway collection containing 262 pathways was obtained from WikiPathways (http://www.wikipathways.org, see Additional file 2). An overrepresentation analysis was performed using differentially expressed genes. The pathways are ranked based on a standardized difference score (z-score) using the expected value and standard deviation of the number of differentially expressed genes in a pathway under a hypergeometric distribution. A positive z-score indicates pathways with a greater number of significantly changed genes than is expected by chance [15]. Pathways were considered significantly changed when (1) Z-score > 1.96, (2) permutated p-value < 0.05 and (3) minimum number of changed genes is 3. Additionally, the log2FC and p-value were visualized on pathways with the visualization module in PathVisio.

### Network analysis

First, all significantly changed pathways were combined and visualized with Cytoscape 3.1.1. The pathways were loaded as networks using the WikiPathways app [16] and an identifier mapping step was performed with the BridgeDb App [17] to unify the identifiers to Ensembl for gene products and HMDB for metabolites. By applying the network merge functionality in Cytoscape the pathways were combined into one integrated network.

Second, the gene expression data was visualized on the nodes of the network. Third, the integrated network was extended with information on transcriptional regulation derived from the ENCODE project [18] using the CyTargetLinker app [19]. Finally, drug-target interactions from DrugBank version 4 [20] were integrated in the network.

The comprehensive visualization functionality in Cytoscape was applied to further explore the complex regulatory mechanisms.

### Workflow

**Figure 1:**
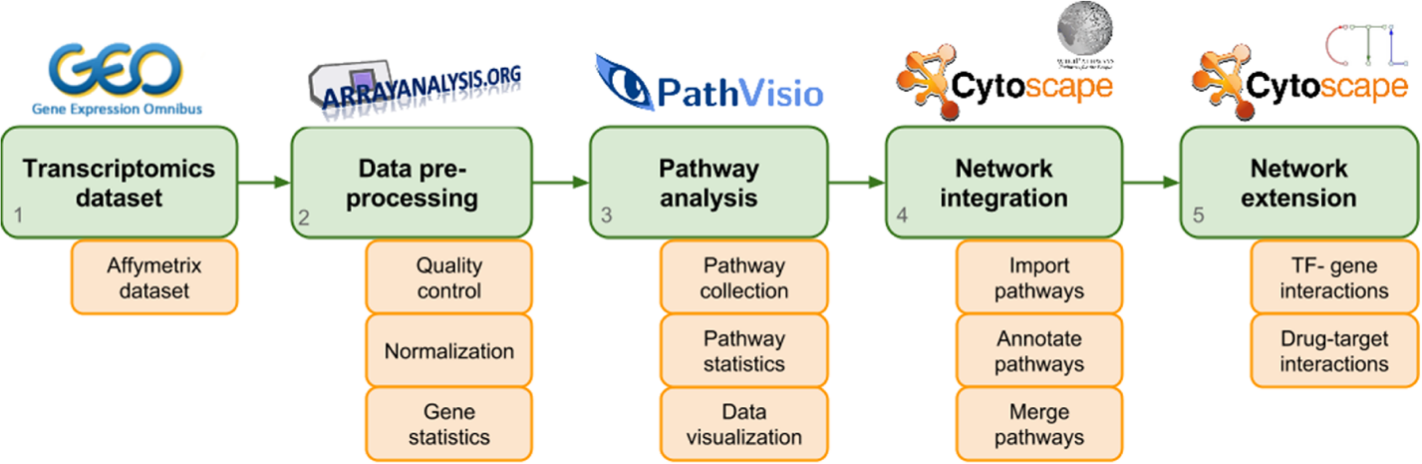
**Transcriptomics network biology workflow.** The described workflow uses four different tools that can be easily connected by taking the output of one tool and importing it in the next tool. No intermediate steps are required.

The strength of the described workflow is that the tools can be easily combined and used together. Figure 1 shows the different steps in the workflow and in each step the output of one tool can be used as the input of the next one. The raw Affymetrix sample files were downloaded from GEO and directly uploaded to ArrayAnalysis.org. This pipeline performs quality control, normalization and statistical analysis of microarray data. The result of the statistical analysis can be used as input for PathVisio without any intermediate steps. The outcome of the pathway statistics in PathVisio is a list of significantly changed pathways. The pathways are stored in GPML format and can be opened in Cytoscape using the WikiPathways app. Using the BridgeDb app, the identifiers of the pathways can be unified if necessary and the Cytoscape merge functionality is used to create one large network from the separate pathways. The CyTargetLinker app then enables the user to extend the network with regulatory interactions like drug-target or TF-gene interactions. A comprehensive description of the workflow can be found on http://projects.bigcat.unimaas.nl/netbio-workflow/.

## Results

A network biology workflow was designed to decipher the biological processes involved in the human diabetic liver. The results obtained with pathway and network analysis will be explained in more detail.

### Differential expression

In the selected human diabetic fatty liver dataset 11,878 genes were measured and annotated in both lean and obese, diabetic subjects. Statistical analysis showed that 181 genes were differentially expressed (absolute logFC > 1 and p-value < 0.05), of these were 118 up-regulated and 63 down-regulated in the obese, diabetic subjects compared to the lean control group. 68 of the up-regulated and 17 of the down-regulated genes have known functions in biological pathways (see Additional file 3: Tables S1 and S2).

The GO analysis was performed with GO-Elite and showed relevant processes for T2DM being over-represented, e.g. triglyceride metabolic process (GO:0006641), cholesterol metabolic process (GO:0008203), glucose metabolic process (GO:0006006), response to glucose stimulus (GO:0009749), cholesterol homeostasis (GO:0042632) and complement activation, classical pathway (GO:0006958). Furthermore, processes related to onecarbon metabolism, humoral immune response, protein-lipid complex subunit organization and organic anion transport were found.

### Pathway analysis

**Figure 2:**
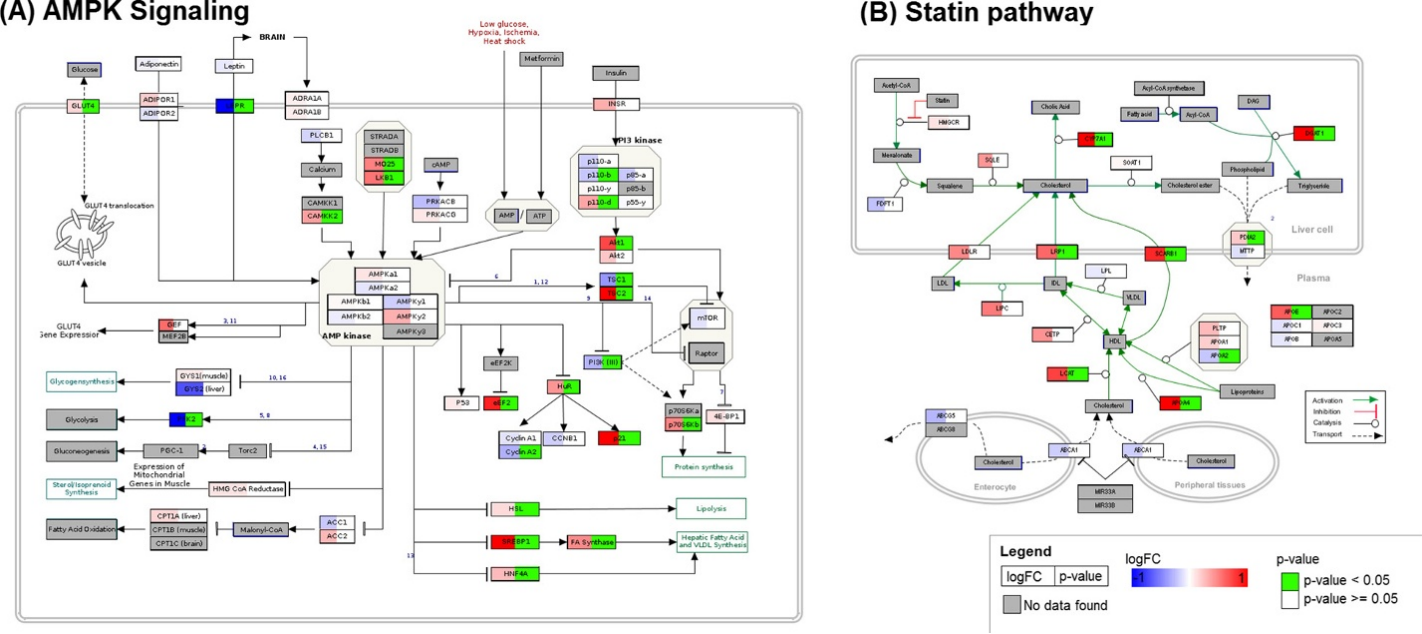
**Visualization of two pathways relevant for drug treatment of T2DM.** Gene expression is visualized on **(A)** AMPK Signaling pathway, http://www.wikipathways.org/instance/WP1403 and **(B)** Statin pathway, http://www.wikipathways.org/instance/WP430 from WikiPathways. The visualization of the gene product boxes in the pathways is split into two parts, (1) the log2 FC in the left part of the box (blue is down-regulated over white is not changed to red is up-regulated) and (2) the p-value in the right part of the box (green when significant). Pathway elements including metabolites that have not been measured in the selected dataset are gray.

Biological processes in which differentially expressed genes are enriched were identified by performing pathway analysis in PathVisio. The statistical analysis resulted in seven significantly changed pathways (Z-score > 1.96, p-value < 0.05, minimum of three changed genes) (see Table 1). Most of these pathways are processes relevant for T2DM but there are also some bigger pathways included, like Proteasome Degradation or Adipogenesis. To illustrate the visualization of the analyzed gene expression data two pathways known to be important in drug treatment of T2DM were selected, i.e. the AMPK Signaling and the Statin pathway (Figure 2A and 2B). Images of all altered pathways are provided in Additional file 4: Figure S1–S7.

**Table 1:** **Seven pathways changed in the diabetic liver**

Pathway	Z-score	P-value	# Genes	Genes
Triacylglyceride Synthesis	3.78	0.001	3 / 19	*↑* AGPAT2, GPD1, DGAT1
Proteasome Degradation	3.32	0.006	5 / 53	*↑*RPN1, PSMB3, HLA-B, HLA-E, HLA-J
Statin Pathway	3.10	0.006	3 / 25	*↑*DGAT1, APOA4, CYP7A1
Fluoropyrimidine Activity	2.84	0.013	3 / 28	*↑*SLC22A7
				*↓*ABCG2, DPYD
Pathogenic Escherichia coli infection	2.76	0.011	4 / 46	*↑* ARPC1A, ARPC1B, ACTB
				*↓* ROCK1
Adipogenesis	2.41	0.016	7 / 121	*↑* SREBF1, CDKN1A, NR1H3, PNPLA3, AGPAT2
				*↓* CISD1, ZMPSTE24
AMPK Signaling	2.38	0.029	4 / 54	*↑* SREBF1, P21
				*↓* LEPR, PFKFB3

Pathway statistics in PathVisio revealed seven significantly altered pathways (Z-score > 1.96, P-value < 0.05, minimum of 3 changed genes). The number of genes (# Genes) represent the number of differentially expressed genes in the pathway compared to the total number of measured genes in the pathway. The arrows indicate up *↑* and down-regulation *↓*.

In the AMPK Signaling pathway the gene expression of the upstream regulating kinases of AMPK, i.e, CAMKK, LKB1, MO25 and STRADA, were significantly up-regulated. Moreover, the expression of the glucose transport protein 4 (GLUT4) is significantly increased together with an increase in the GLUT4 enhancer factor (GEF; p-value = 0.057). Most downstream AMPK targets were up-regulated, i.e., HNF4A, SREBF1, eEF2, TSC2, p21 and some are down-regulated, like PFK2 and TSC1.

In the Statin pathway the inhibitory action of statin, a cholesterol-lowering drug, on HMG-CoA reductase (HMGCR) is depicted. The expression of HMGCR remains unaltered in the diabetic fatty liver compared to lean controls. Moreover, cholesterol synthesis is described in the pathway and almost all differentially expressed genes are up-regulated, like DGAT1, CYP7A1, SCARB1, LCAT and APOA4.

### Network analysis

#### Pathway integration

Pathway analysis revealed seven pathways with a Z-score > 1.96 (see Table 1) which were then combined into one biological network and analyzed in the network visualization and analysis tool Cytoscape. The created network contains 642 edges connecting 580 nodes, consisting of 408 gene products, 38 metabolites and 5 pathway nodes. 129 nodes are visualized as very small nodes to represent groups and complexes as well as complex interactions in the pathways. Pathways from WikiPathways can contain pathway nodes that link to other pathways. The created network has therefore links to five other pathways: Glycolysis (WP534), DNA Repair (WP1805), Fatty Acid Oxidation (WP143), Fatty Acid Synthesis (WP357) and Apoptosis (WP254).

A figure of the complete network is available in Additional file 5: Figure S8. Fourteen genes in the network are significantly up regulated in obese, diabetic subjects including three genes linking two or more pathways such as AGPAT2, CDKN1A and SREBF1. Seven genes, all present in only one pathway, are significantly down regulated.

**Figure 3:**
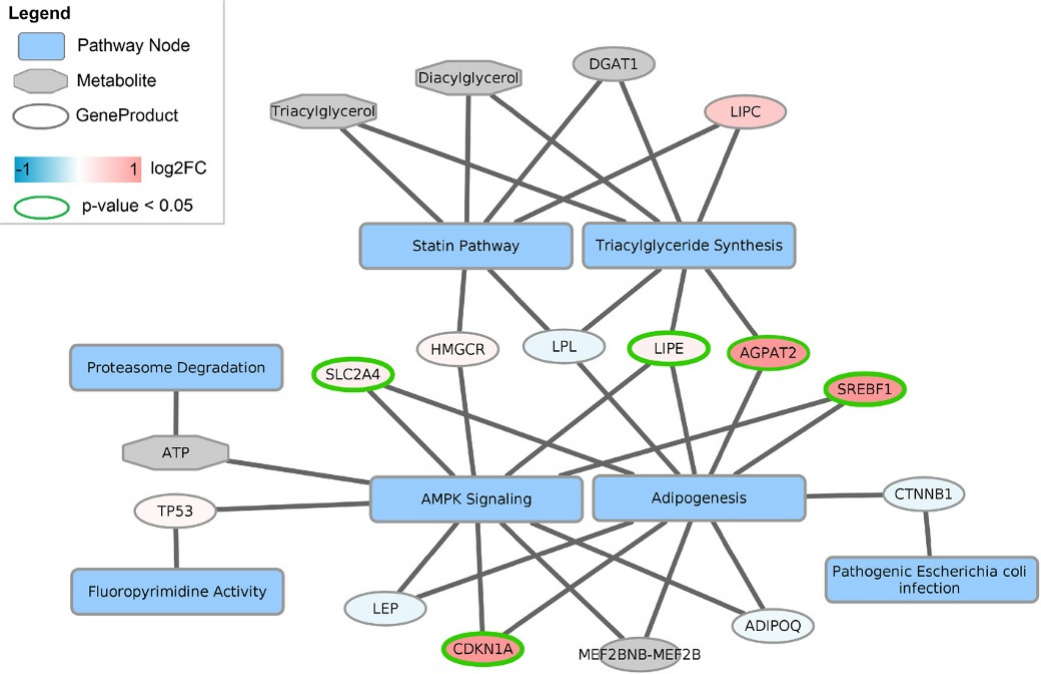
**Nodes linking the seven significantly changed pathways.** Each pathway is represented as a yellow rounded rectangle. Gene products and metabolites are visualized as ellipses and octagons, respectively. The transcription dataset is visualized on the gene nodes in the network using a color gradient from blue (down-regulated) over white (not changed) to red (up-regulated). Nodes with a significant p-value (< 0.05) have a light-green border color. Most nodes linking multiple pathways are either up-regulated (e.g. SREBF1, CDKN1A, AGPAT2) or not altered significantly (e.g. LPL, HMGCR).

Figure 3 shows how fourteen genes and three metabolites are linking two or more of the selected pathways to each other. The transcriptomics dataset comparing obese, diabetic subjects with lean subjects is visualized in the network. The log2FC is indicated by a color gradient on the nodes and significance (p-value < 0.05) is represented by a light-green border. Nodes including metabolites without a measurement in the dataset are colored gray.

In the liver of obese, diabetic subjects, the gene expression of three of the linker genes, i.e. AGPAT2, CDKN1A and SREBF1, is significantly (p < 0.05) up-regulated. AGPAT2 is an enzyme that plays an important role in the production of glycerophospholipids and triacylglycerols. It is known to be relevant to the liver and development of hepatic steatosis [21]. CDKN1A is a potent cell cycle inhibitor important for the induction and maintenance of cellular senescence. SREBF1 is a TF regulating genes required for glucose and fatty acids metabolism and lipid synthesis. Studies showed a clear link between mutations in CDKN1A and SREBF1 and the risk of developing NAFLD [22, 23] strengthening the involvement of these genes in diabetic fatty liver. A table describing the function, gene expression and their involvement in NAFLD and diabetes for all linking genes is provided in Additional file 6: Table S3.

#### Extension with transcriptional regulation

The integrated network was extended with transcriptional regulation to obtain a better insight in how biological processes affected in the human fatty liver are regulated. The CyTargetLinker app in Cytoscape was used to extend the network with proximal TF-target interactions from the ENCODE project [24]. The app identified sixteen nodes in the network as TFs, most of which are nodes present in only one pathway, except for SREBF1. All TFs are present in either the AMPK Signaling pathway or the Adipogenesis pathway.

**Figure 4:**
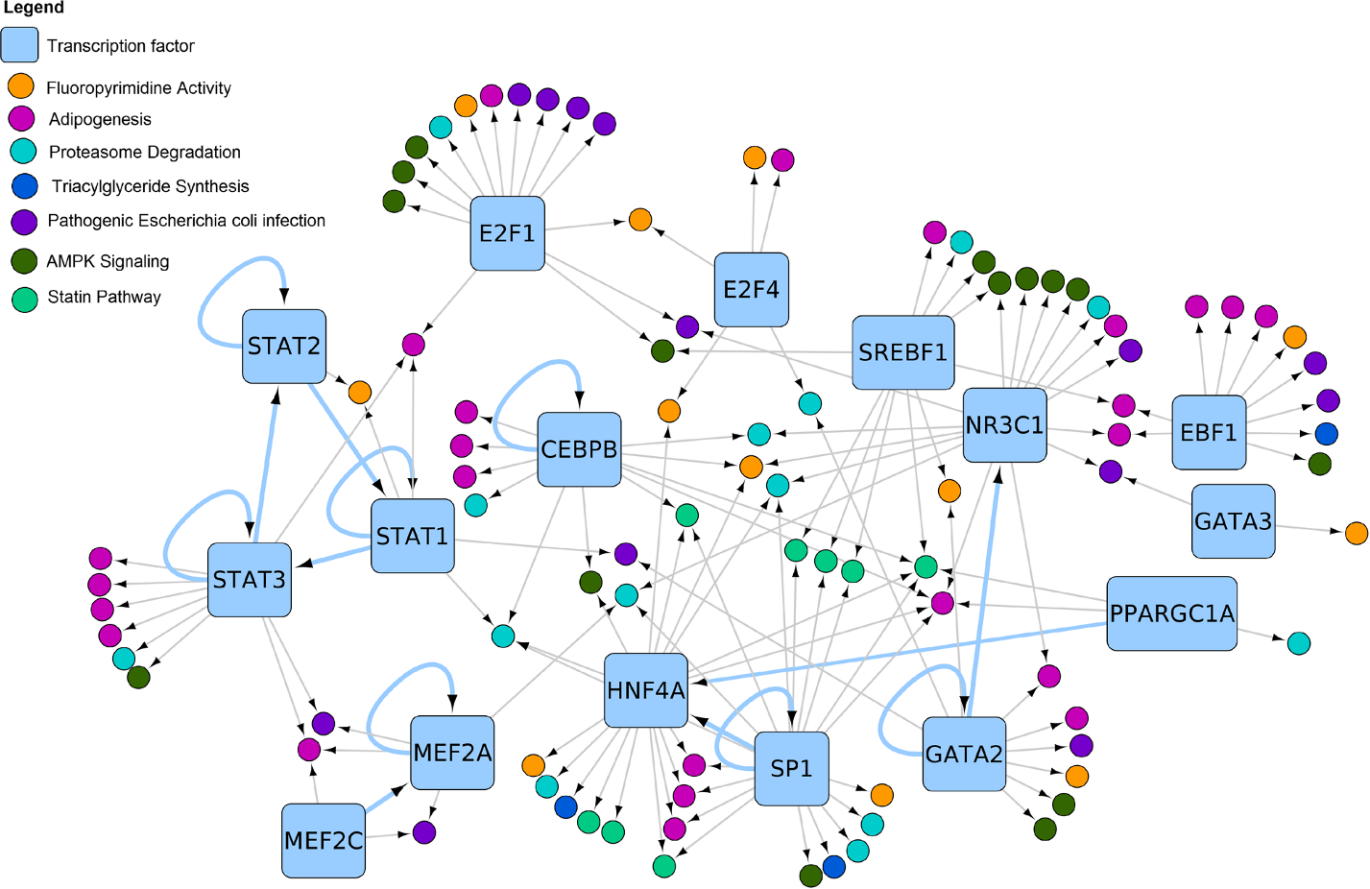
**TF regulation in the diabetic fatty liver pathways.** Using CyTargetLinker, sixteen TFs have been identified in the seven pathways. TFs are visualized as rounded rectangles and their target genes as circles colored based on their presence in different pathways. 56 genes are targeted by only one TF and 33 genes are targeted by 2 or more TFs. Light-blue edges indicate regulation of TFs by other TFs.

Figure 4 shows a network containing the sixteen TFs as diamonds and 90 of their targets which are present in one of the selected seven pathways. The interactions in this network are not present in the pathways but have been reported in the ENCODE regulatory network derived by Gerstein *et al.* [18]. In the initial network, pathway elements were not present in more than three out of seven pathways, however with the CyTargetLinker app, TFs were identified that target genes in up to six out of seven different pathways, like SP1 or HNF4A. This approach also found some TFs regulating other TFs (highlighted as light-blue edges) and discovered typical network motifs like feed-forward loops (e.g. STAT1 → STAT3 → STAT2 → STAT1) or self-regulation (e.g. SP1, GATA2 or CEBPB). Three of the TFs in the network are up-regulated in obese, diabetic subjects, SREBF1 (log2FC: 1.05, p-value: 0.03), STAT3 (log2FC: 0.88, p-value: 0.008) and CEBPB (log2FC: 0.41, p-value: 0.01). All three have been reported to play a role in the development of NAFLD [25–27].

#### Extension with drug-target information

**Figure 5:**
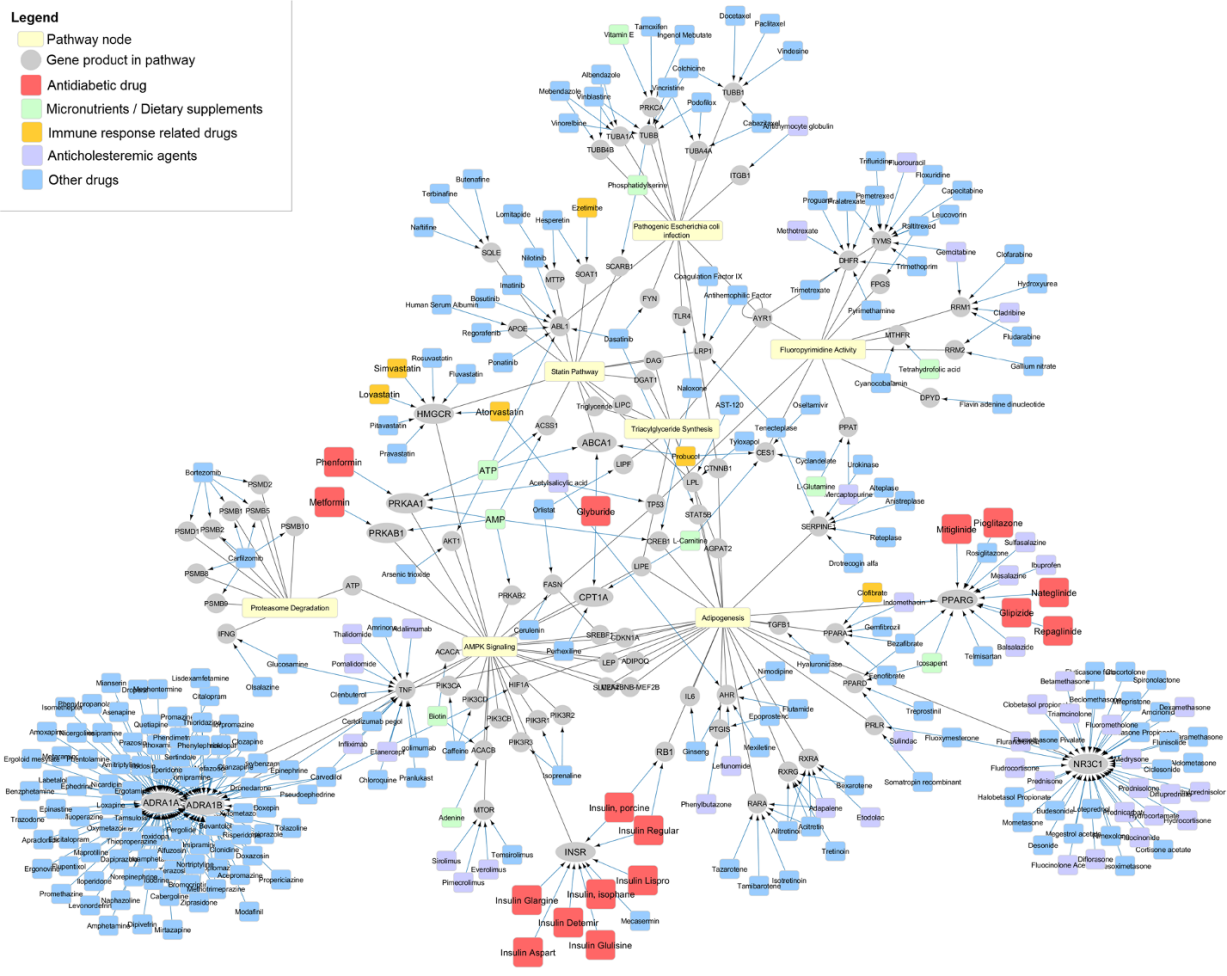
**Significant amount of antidiabetic drugs targeting gene products in the network.** The network has been extended with drug-target interactions from DrugBank 4 using CyTargetLinker. Nodes present in only one pathway and not targeted by any drugs have been grouped in pathway nodes (yellow rounded rectangles). Drugs targeting genes in the network are indicated as blue rectangles, drugs associated with diabetes are colored in red, micronutrients/dietary supplements in green, drugs related to immune response in orange and anticholesteremic agents in purple. Diabetes related drugs target 7 gene products in the network: INSR (8 drugs), PPARG (5 drugs), RB1 (2 drugs), ABCA1 (1 drug), CPT1A (1 drug), PRKAA1 (1 drug), PRKAB1 (1 drug).

CyTargetLinker provides a regulatory interaction network for drug-target interactions from DrugBank. The combined network was extended only with approved drugs leaving out the ones that are withdrawn or experimental. In total 280 drugs were added targeting 76 gene products in the pathways, see Figure 5. Based on the categories used in DrugBank the drugs associated with the treatment of diabetes (= antidiabetic and hypoglycemic agents; colored in red), dietary supplements/micronutrients (colored in green), immune response related (colored in orange) and anticholesteremic agents (colored in purple) are highlighted in Figure 5. In the extended network, the amount of drug targets related to diabetes is significantly higher than random (see Additional file 7: Figures S9 and S10).

The insulin receptor (INSR) is targeted by nine drugs of which eight are categorized in DrugBank as antidiabetic and/or hypoglycemic agents and the ninth, Mecasermin, is an insulin-like growth factor used for long-term treatment of growth failure in children with severe primary IGF-1 deficiency [28]. The INSR is activated by insulin binding and after activation it phosphorylates and thereby activates insulin receptor substrate 1 (IRS1) which in turn activates the PI3-kinase and AKT signalling pathways. Insulin is a natural hormone produced by beta cells in the pancreas and has many functions, e.g. promotion of cellular uptake of glucose and energy storage via glycogenesis. T2DM patients who are unable to control their glucose levels can be treated with insulin analogues, like Insulin Aspart, Detemir or Glargine and these are indeed drugs targeting the INSR in the drug-target network.

Furthermore, two diabetes related drugs, Phenformin and Metformin, target the two subunits of AMPK, PRKAA1 and PRKAB1. Both subunits are also activated by a member of the adenine nucleotide family, adenosine monophosphate (AMP), which by DrugBank is categorized as dietary supplement and micronutrient. The network shows that several hypoglycemic agents, like Rosiglitazone, Glipizide or Pioglitazone, are known to target PPARG, a receptor which regulates fatty acid storage and glucose metabolism. All these drugs are known to be prescribed to T2DM patients depending on the state of the disease.

## Discussion

It has been shown that insulin resistance conditions like obesity and T2DM are strongly associated with the accumulation of lipids in the liver, which is one of the main characteristics of NAFLD [29, 30]. Although it is known that impaired substrate metabolism is involved in the development of the fatty liver in T2DM, the exact mechanisms remain unclear. In this study, we applied a network biology approach to investigate the molecular mechanisms in the diabetic fatty liver.

Biological pathway information is useful for better understanding the mechanisms affected by disease. As illustrated in this study, researchers can investigate the pathways in diseased subjects compared to control subjects to gain more insights into the causes of a disease and the mechanisms of disease progression. Although pathway collections cover many well-described biological mechanisms, they are incomplete, resulting in a bias towards well-studied processes. Nevertheless, pathway databases are growing and pathway analysis has proven itself as a valid and intuitive first step in the analysis process. Wiki-based community curated pathway databases like WikiPathways reduce the barrier to participation in pathway curation and allow experts to add new findings immediately to the pathway diagrams.

While standard pathway analysis investigates each pathway individually, biological processes are not independent but rather interact with and influence each other. Therefore it is relevant to investigate the links between them as well as shared regulatory mechanisms. This study describes a workflow for further exploring the interplay between pathways involved in the human fatty liver by connecting them in a biological network and extending them with additional knowledge on TF regulation and drug targeting.

Pathway analysis demonstrated that Triacylglyceride Synthesis and Adipogenesis are significantly altered in obese, diabetic subjects with a fatty liver compared to the lean control group. The pathway analysis was performed using gene expression measured in liver biopsies. Although the biopsies mostly consist of hepatocytes also other cells like immune, vascular and fat cells are present. Interestingly, two pathways related to drug treatment in T2DM (i.e. the AMPK signaling pathway and the Statin pathway) are among the significantly altered pathways.

AMPK is known as the metabolic regulator, and its activation influences many metabolic processes [31]. Under catabolic situations, like in a fasted state, AMPK is activated, thereby increasing glycolysis and FA betaoxidation and decreasing FA synthesis [32]. AMPK activation requires both AMP binding and phosphorylation in the catalytic alpha subunit. Phosphorylation of AMPK is accomplished by the upstream regulators, CAMKK2 and LKB1 in complex with MO25 and STRADA [33]. The gene expression of all upstream regulators of AMPK are significantly up-regulated showing that the AMPK pathway is over-represented in the human diabetic liver.

In the Statin pathway, the expression of genes involved in cholesterol and triacylglycerol production (DGAT1, CYP7A1, SCARB1, LCAT and APOA4) are all significantly up-regulated in the diabetic liver in humans. These findings indicate that hepatic lipid accumulation is facilitated by an increased expression of these genes.

Regulatory elements like TFs are often not included in pathway diagrams in order to keep the diagrams comprehensible. Nevertheless, as this study confirms, TFs play a crucial role in the understanding of complex diseases since they often regulate multiple pathways simultaneously. Our analysis shows that TFs can be considered as additional links between pathways, and adding the regulatory interactions increases the overall connectivity of the network significantly. Typical TF network motifs (e.g. feed-forward loops, single input module or self-regulation) can be identified using standard network algorithms.

Hepatocyte nuclear factor 4-alpha (HNF4A) is an essential TF in the extended regulatory network (see Figure 4) which is regulated by two TFs (i.e. PPARGC1A and SP1) and regulates genes in 6 out of 7 pathways (not in the E.coli infection pathway). HNF4A is a nuclear receptor (NR), which is a key regulator of the liver cell function, is a sensor of inflammation and is known to regulate genes in lipid and glucose metabolism [34].

Furthermore, CyTargetLinker revealed 93 additional TFs, not yet present in one of the pathways, which target 212 nodes in the network. TFs generally regulate multiple targets, and thus more than 800 regulatory interactions have been added. Two hub TFs targeting more than 35 genes in the pathways are CTCF and EP300. CTCF is a general TF that has been reported to mediate the effects of insulin on glucagon expression and therefore is a possible new target for diabetes treatment [35]. EP300 is a general TF that regulates cell growth and division and has been reported as a key participant in hepatic steatosis [27].

The extension of the network with drug-target information resulted in a higher number of known drugs used to treat T2DM than expected which confirms the validity of the approach described in this study. The network can be used to show where drug targets are located and what effect can be expected in a pathway. It might be possible to identify other drugs with similar pharmacological effects or advantageous drug combinations when targeting two positions in the network to get a stronger effect of the treatment, e.g. combination of Glipizide (PPARG) and Metformin (AMPK). It might also be used to identify new drugs targeting pathway elements upstream, downstream or even parallel to the currently used targets. When studying the effects of drugs on a pathway, the grouped pathway nodes in Figure 5 can be further expanded to view the complete pathway and its interactions.

Besides the diabetes related drugs, there are many other drugs known to target genes in the selected pathways. HMGCR, a highly targeted gene product, is part of the Statin pathway depicted in Figure 2A and is targeted by seven drugs, all belonging to the statin family. This drug family has a cholesterol lowering effect and is often used in combination with anti-diabetic drugs [36]. Furthermore, three gene products in the network (ADRA1A, ADRA1B and NR3C1) are highly targeted by more than 40 drugs. ADRA1A and ADRA1B are members of a subfamily of the G protein-coupled receptors (GPCRs), and there are several studies investigating the potential of GPCRs for the treatment of T2DM [37, 38]. NR3C1 is part of the NR superfamily of TFs that are known to play a role in the development and adaptations of liver diseases. NRs are also suggested as potential drug targets for the treatment of diabetes and NAFLD [39].

In general, the described workflow can be applied to different datasets, diseases and pathway collections. It also allows the analysis of multiple datasets together by performing separate pathway analyses and then combining the pathway results in one integrated network. The integration of pathway information into the network allows researchers to investigate the downstream effects of drugs and contributes to the identification of other treatment possibilities. Also, the link with other pathways that are affected or not affected by the disease state is important for predicting possible side effects of a drug. Including the information about the effects of other drugs in linked or related pathways can help to identify potential interferences with the efficacy and efficiency of a drug.

## Conclusions

In this study we demonstrated how pathway analysis results, which are often considered a final step in the biological interpretation of transcriptomics data, can be used and combined in a biological network to gain more insights in the interplay and relation between processes. Instead of starting with a large protein-protein interaction network and finding the important parts in it, we believe that building the networks based on relevant pathways can be another very useful approach to start the investigation. Also the inclusion of all elements present in the pathway provides a framework which can integrate different types of omics-data, like omics techniques applied to any gene product (transcriptomics, proteomics, etc) as well as metabolomics or fluxomics. The biological interpretation might be more straight-forward because it builds on the pathway diagrams which are usually intuitive and well studied.

Regulation by TFs or drugs does not only have effects on one pathway but also has effects on downstream processes. This integration leads to improved insight and also a much clearer illustration of the overall process, and the most important elements. Inclusion of information about drugs and micronutrients and their targets makes the mode of action of currently used compounds more understandable and can be useful to suggest drug repositioning and new drugs or micronutrient related lifestyle interventions.

The tools used in this study, especially PathVisio, Cytoscape and CyTargetLinker, facilitated the data integration, visualization and interpretation immensely.

## Funding

This work was (co)financed by the Netherlands Consortium for Systems Biology (NCSB) which is part of the Netherlands Genomics Initiative/Netherlands Organisation for Scientific Research (http://www.ncsb.nl).

## Electronic supplementary material

Additional file 1: **Quality control report provided by ArrayAnalysis.org before and after normalization.** (PDF 1 MB)

Additional file 2: **WikiPathways human pathway collection downloaded on 17 June 2014 (** http://wikipathways.org/index.php/Download_Pathways **).** (ZIP 2 MB)

Additional file 3: **Table S1.** 118 upregulated genes in obese, diabetic patients with a fatty liver. **Table S2.** 63 downregulated genes in obese, diabetic patients with a fatty liver. (PDF 62 KB)

Additional file 4: **Figure S1–S7.** Images of all seven altered pathways with dataset visualized on the pathways. (PDF 452 KB)

Additional file 5: **Figure S8.** Integrated network of seven interconnected pathways that are changed in the diabetic fatty liver. (PNG 1 MB)

Additional file 6: **Table S3.** List of genes present in two or more pathways. (PDF 35 KB)

Additional file 7: **Figure S9.** Drug extension simulation for all drugs. **Figure S10.** Drug extension simulation for antidiabetic drugs. (PDF 59 KB)

## Abbreviations

T2DM: Type 2 diabetes mellitus
NAFLD: Non-alcoholic fatty liver disease
TF: Transcription factor
FC: Fold change
GO: Gene ontology
NR: Nuclear receptor
GPCRs: G protein-coupled receptors.
