# Supplementary material for: A network biology workflow to study transcriptomics data of the diabetic liver
Source: BMC Genomics. 2014 Nov 15;15(1):971. doi: 10.1186/1471-2164-15-971 (PMC4246458; doi:10.1186/1471-2164-15-971)
Supplement: Supplementary file 1 — Additional file 1: Quality control report provided by ArrayAnalysis.org before and after normalization. (PDF 1 MB) [file 12864_2014_6667_MOESM1_ESM.pdf]

# Array names and grouping

| ArrayDataFile | SourceName | FactorValue |
|---|---|---|
| GSM391693.CEL | Liver_Lean_rep1 | Liver_Lean |
| GSM391694.CEL | Liver_Lean_rep2 | Liver_Lean |
| GSM391695.CEL | Liver_Lean_rep3 | Liver_Lean |
| GSM391696.CEL | Liver_Lean_rep4 | Liver_Lean |
| GSM391697.CEL | Liver_Lean_rep5 | Liver_Lean |
| GSM391702.CEL | Liver_Obese_DM_rep1 | Liver_Obese_DM |
| GSM391703.CEL | Liver_Obese_DM_rep2 | Liver_Obese_DM |
| GSM391704.CEL | Liver_Obese_DM_rep3 | Liver_Obese_DM |
| GSM391705.CEL | Liver_Obese_DM_rep4 | Liver_Obese_DM |
| GSM391706.CEL | Liver_Obese_DM_rep5 | Liver_Obese_DM |
| GSM391707.CEL | Liver_Obese_DM_rep6 | Liver_Obese_DM |
| GSM391708.CEL | Liver_Obese_DM_rep7 | Liver_Obese_DM |
| GSM391709.CEL | Liver_Obese_DM_rep8 | Liver_Obese_DM |
| GSM391710.CEL | Liver_Obese_DM_rep9 | Liver_Obese_DM |

# Quality Control & Pre-processing Evaluation

of

raw-data_0

# REPORT

# Summary of raw data quality indicators

blue = "within" / red = "out of" recommended cut-off

| | 3'/5' beta-actin (cutoff=3) | 3'/5' GAPDH (cutoff=1.25) | Hybridization BioB<BioC<BioD<CreX | Hybridization BioB=Present | Percent Present spread<=10% | Background spread<=20% | Log Scale Factor spread<=3 |
|---|---|---|---|---|---|---|---|
| Liver_Lean_rep1 | 1.12 | 1.07 | T | P | 35 % | 48 | 1.08 |
| Liver_Lean_rep2 | 1.17 | 1.12 | T | P | 37 % | 49 | 1.36 |
| Liver_Lean_rep3 | 1.19 | 1.11 | T | P | 36 % | 47 | 1.44 |
| Liver_Lean_rep4 | 1.15 | 1.08 | T | P | 34 % | 63 | 0.46 |
| Liver_Lean_rep5 | 1.09 | 1.12 | T | P | 33 % | 60 | 0.79 |
| Liver_Obese_DM_rep1 | 1.15 | 1.07 | T | P | 35 % | 53 | 0.76 |
| Liver_Obese_DM_rep2 | 1.01 | 1.02 | T | P | 36 % | 55 | 0.81 |
| Liver_Obese_DM_rep3 | 1 | 1.02 | T | P | 34 % | 49 | 0.88 |
| Liver_Obese_DM_rep4 | 1.14 | 1.07 | T | P | 35 % | 58 | 0.52 |
| Liver_Obese_DM_rep5 | 1.17 | 1.08 | T | P | 32 % | 68 | 0.52 |
| Liver_Obese_DM_rep6 | 1.01 | 1.05 | T | P | 43 % | 64 | -0.04 |
| Liver_Obese_DM_rep7 | 1.04 | 1.03 | T | P | 38 % | 57 | 0.74 |
| Liver_Obese_DM_rep8 | 1.06 | 1.02 | T | P | 41 % | 54 | 0.09 |
| Liver_Obese_DM_rep9 | 1.03 | 1.04 | T | P | 34 % | 52 | 0.56 |

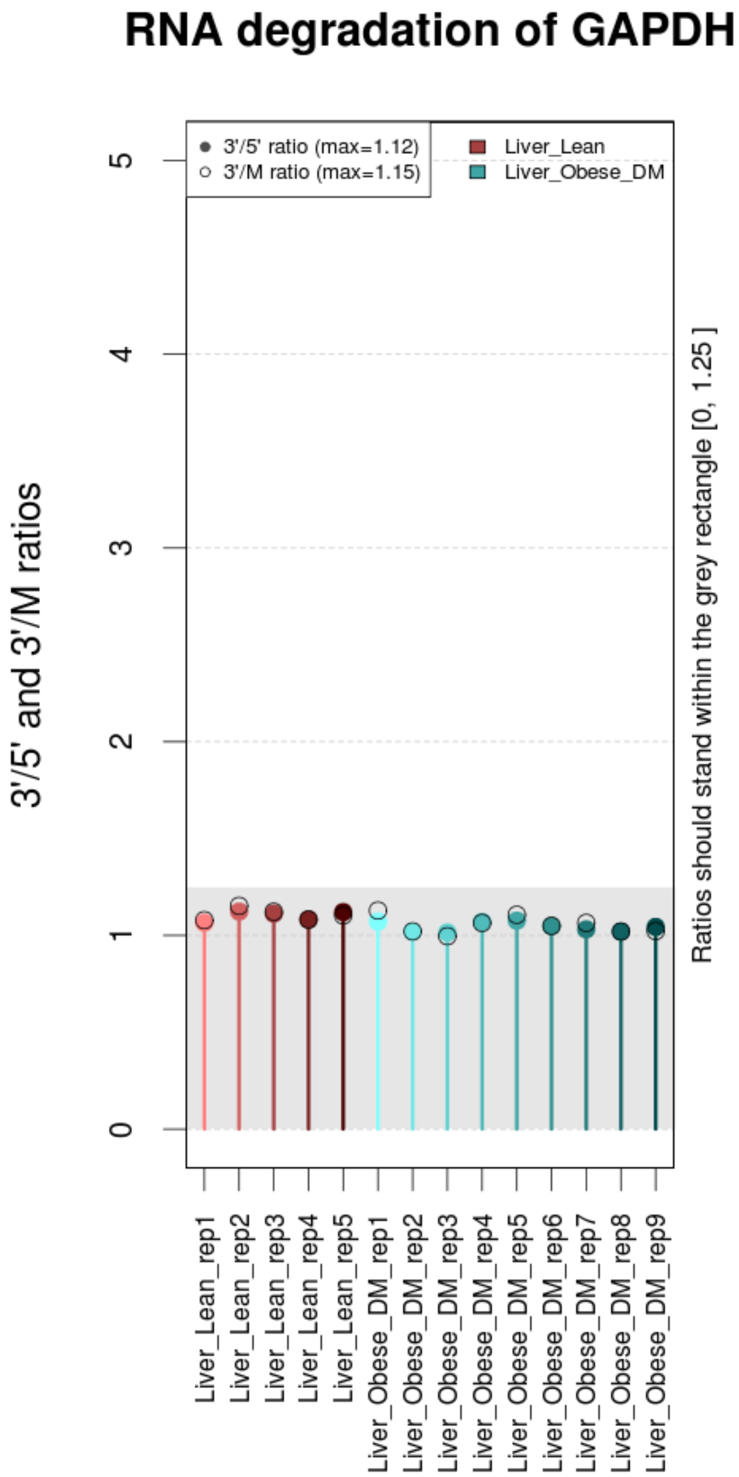
RNA degradation of GAPDH
3'/5' ratio (max=1.12)
3'/M ratio (max=1.15)
Liver_Lean
Liver_Obese_DM
3'/5' and 3'/M ratios
Ratios should stand within the grey rectangle [0, 1.25]
0
1
2
3
4
5
Liver_Lean_rep1
Liver_Lean_rep2
Liver_Lean_rep3
Liver_Lean_rep4
Liver_Lean_rep5
Liver_Obese_DM_rep1
Liver_Obese_DM_rep2
Liver_Obese_DM_rep3
Liver_Obese_DM_rep4
Liver_Obese_DM_rep5
Liver_Obese_DM_rep6
Liver_Obese_DM_rep7
Liver_Obese_DM_rep8
Liver_Obese_DM_rep9

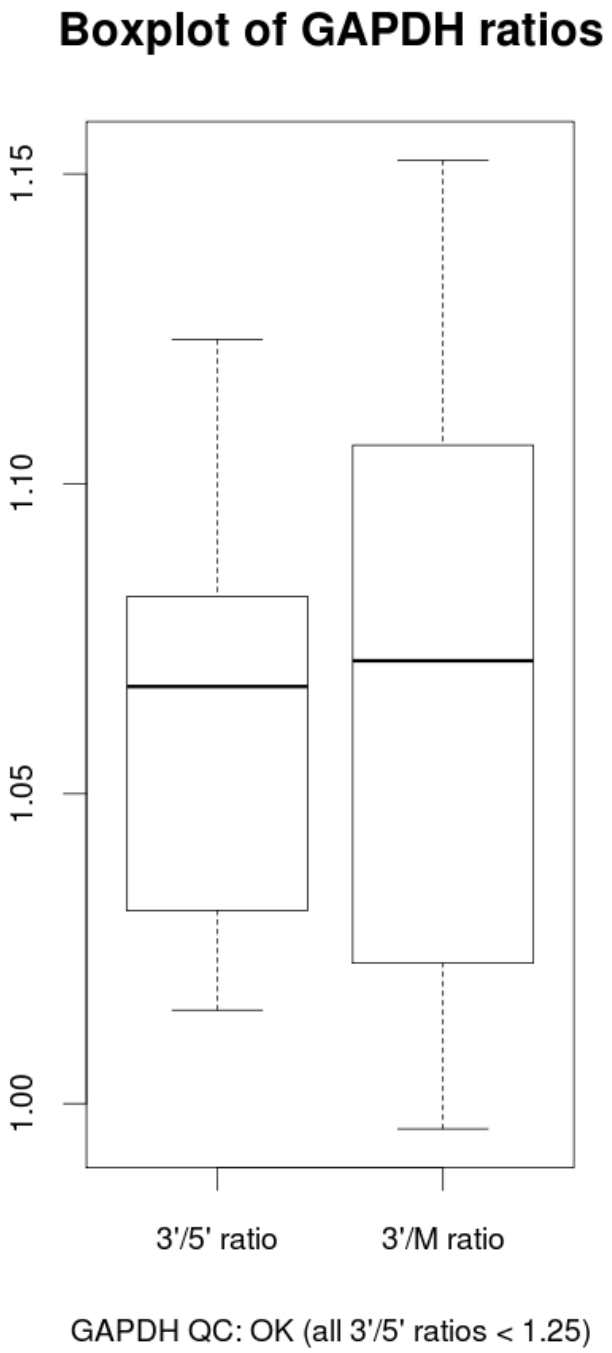
Boxplot of GAPDH ratios
1.00
1.05
1.10
1.15
3'/5' ratio
3'/M ratio
GAPDH QC: OK (all 3'/5' ratios < 1.25)

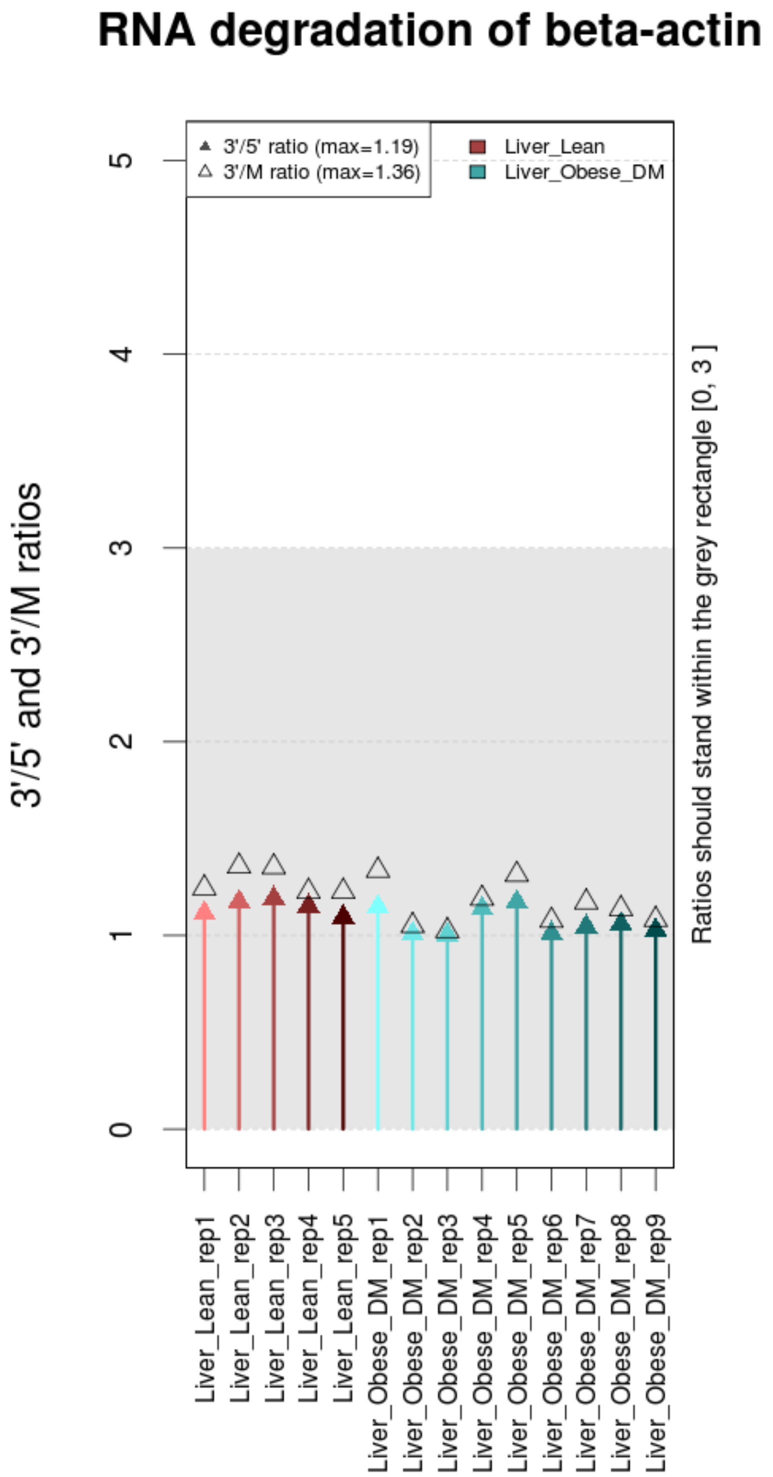
RNA degradation of beta-actin
3'/5' and 3'/M ratios
3'/5' ratio (max=1.19)
3'/M ratio (max=1.36)
Liver_Lean
Liver_Obese_DM
Ratios should stand within the grey rectangle [0, 3 ]
0
1
2
3
4
5
Liver_Lean_rep1
Liver_Lean_rep2
Liver_Lean_rep3
Liver_Lean_rep4
Liver_Lean_rep5
Liver_Obese_DM_rep1
Liver_Obese_DM_rep2
Liver_Obese_DM_rep3
Liver_Obese_DM_rep4
Liver_Obese_DM_rep5
Liver_Obese_DM_rep6
Liver_Obese_DM_rep7
Liver_Obese_DM_rep8
Liver_Obese_DM_rep9

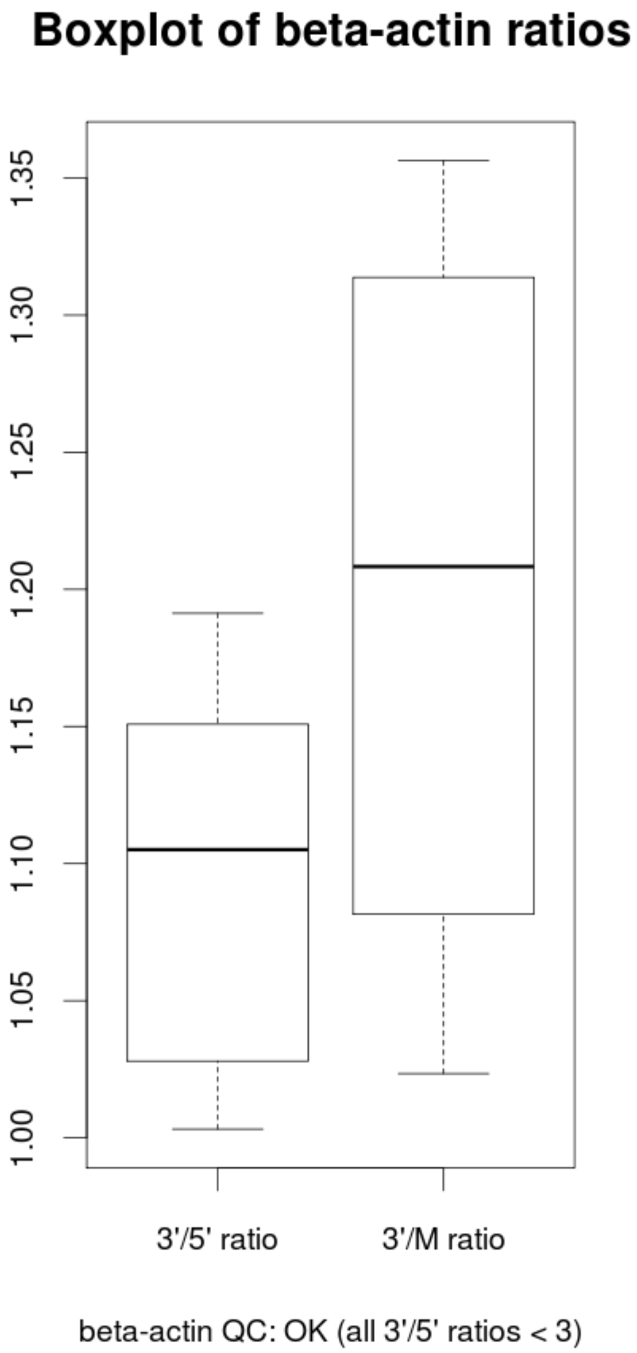
Boxplot of beta-actin ratios
1.00
1.05
1.10
1.15
1.20
1.25
1.30
1.35
3'/5' ratio
3'/M ratio
beta-actin QC: OK (all 3'/5' ratios < 3)

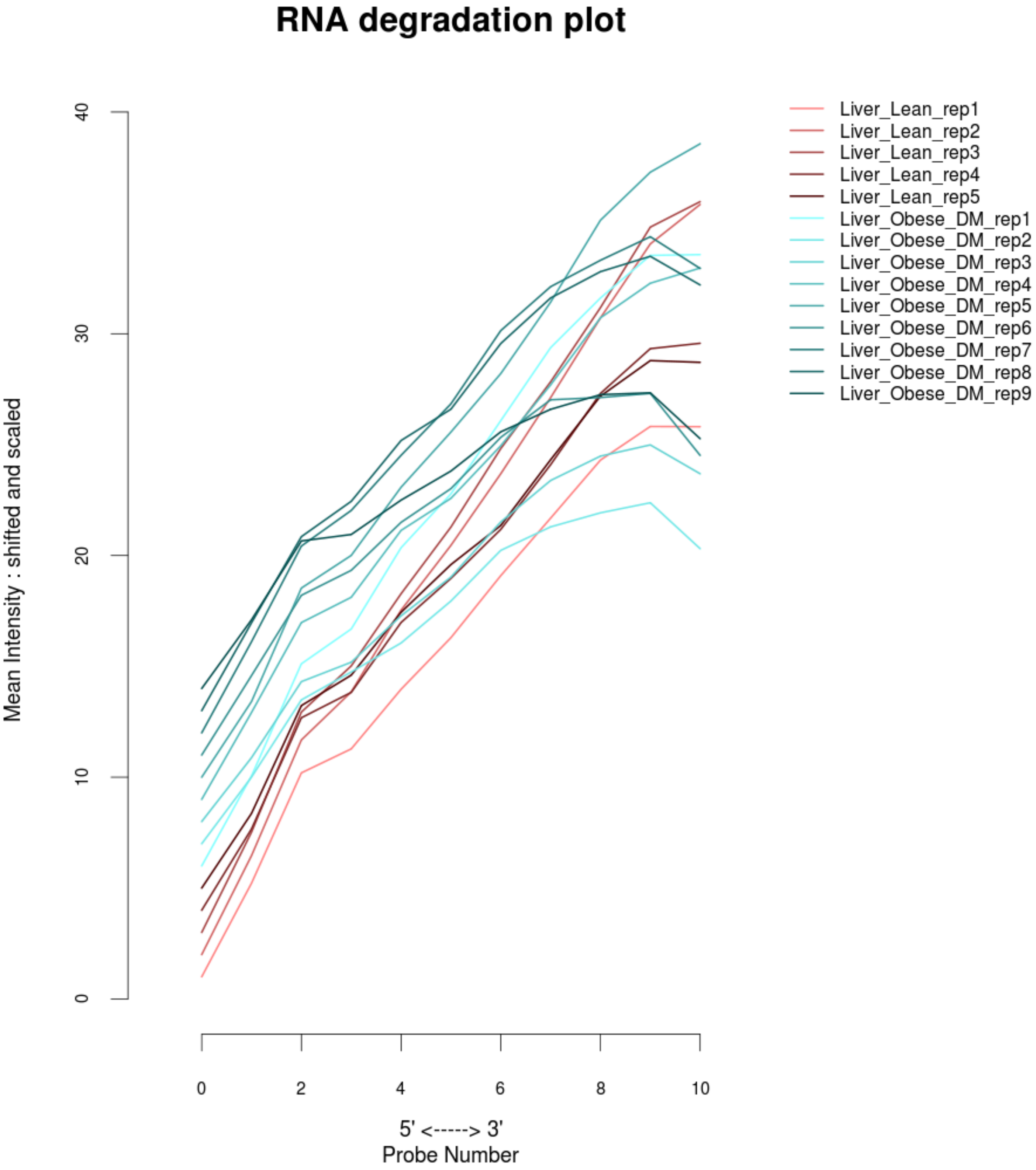

RNA degradation plot
Mean Intensity : shifted and scaled
0
10
20
30
40
0
2
4
6
8
10
5' <-----> 3'
Probe Number
Liver_Lean_rep1
Liver_Lean_rep2
Liver_Lean_rep3
Liver_Lean_rep4
Liver_Lean_rep5
Liver_Obese_DM_rep1
Liver_Obese_DM_rep2
Liver_Obese_DM_rep3
Liver_Obese_DM_rep4
Liver_Obese_DM_rep5
Liver_Obese_DM_rep6
Liver_Obese_DM_rep7
Liver_Obese_DM_rep8
Liver_Obese_DM_rep9

# Spike-in Hybridization controls intensities and calls

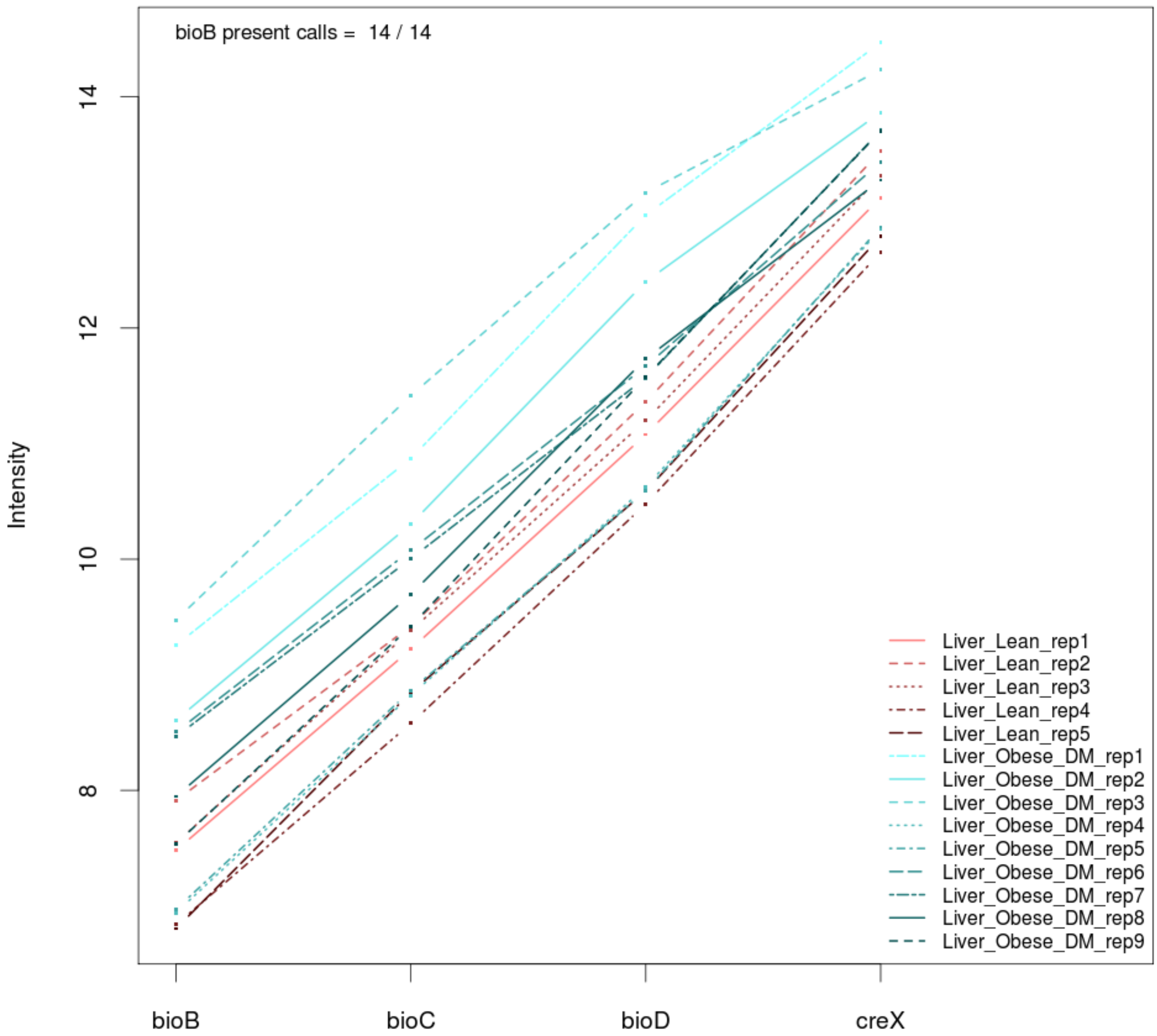


Intensities: OK (bioB < bioC < bioD < creX for all arrays)

BioB Present calls: OK (indeed all bioB are called present)

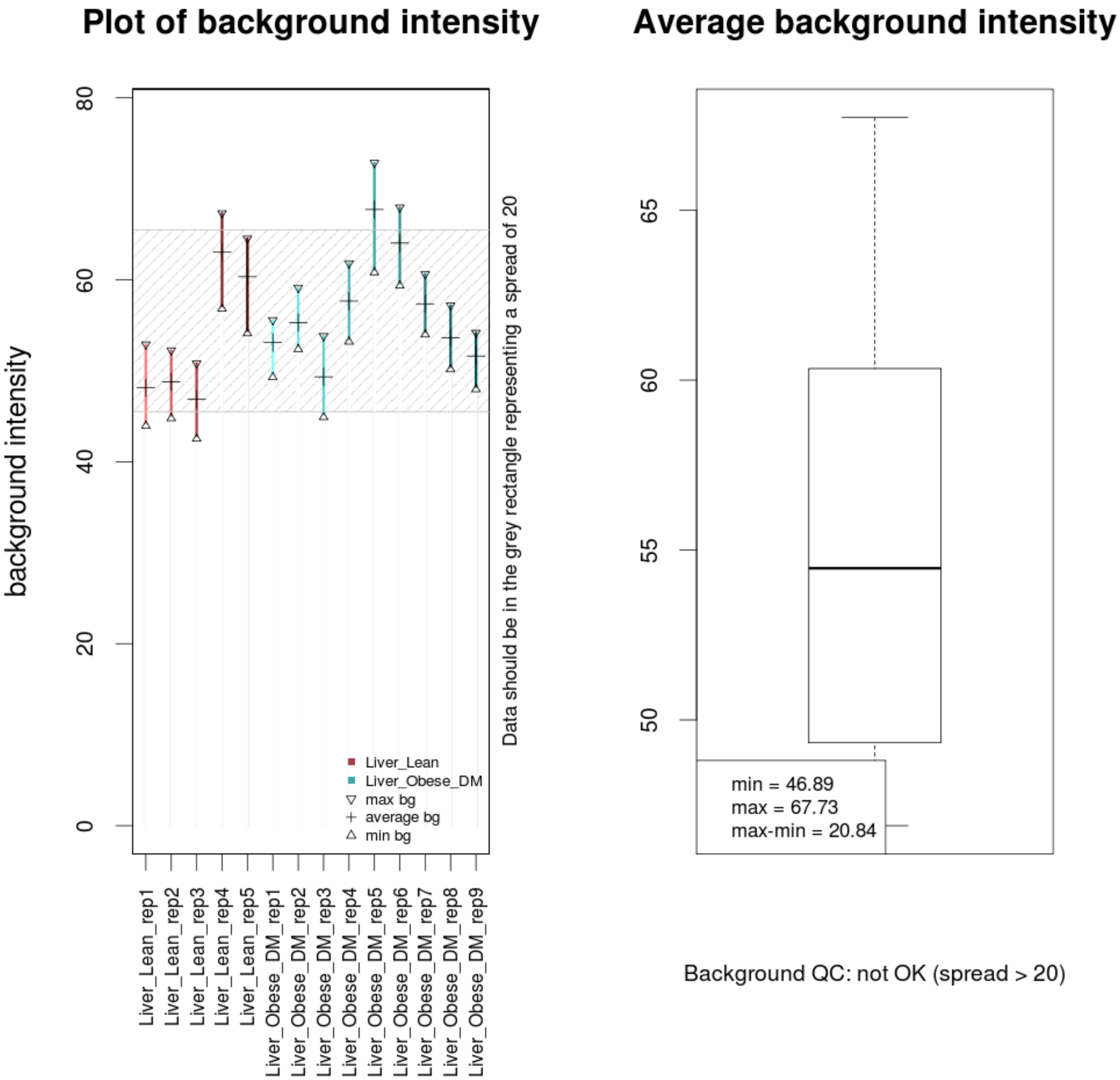

Plot of background intensity
background intensity
0
20
40
60
80
Liver_Lean
Liver_Obese_DM
max bg
average bg
min bg
Data should be in the grey rectangle representing a spread of 20
Liver_Lean_rep1
Liver_Lean_rep2
Liver_Lean_rep3
Liver_Lean_rep4
Liver_Lean_rep5
Liver_Obese_DM_rep1
Liver_Obese_DM_rep2
Liver_Obese_DM_rep3
Liver_Obese_DM_rep4
Liver_Obese_DM_rep5
Liver_Obese_DM_rep6
Liver_Obese_DM_rep7
Liver_Obese_DM_rep8
Liver_Obese_DM_rep9
Average background intensity
50
55
60
65
min = 46.89
max = 67.73
max-min = 20.84
Background QC: not OK (spread > 20)

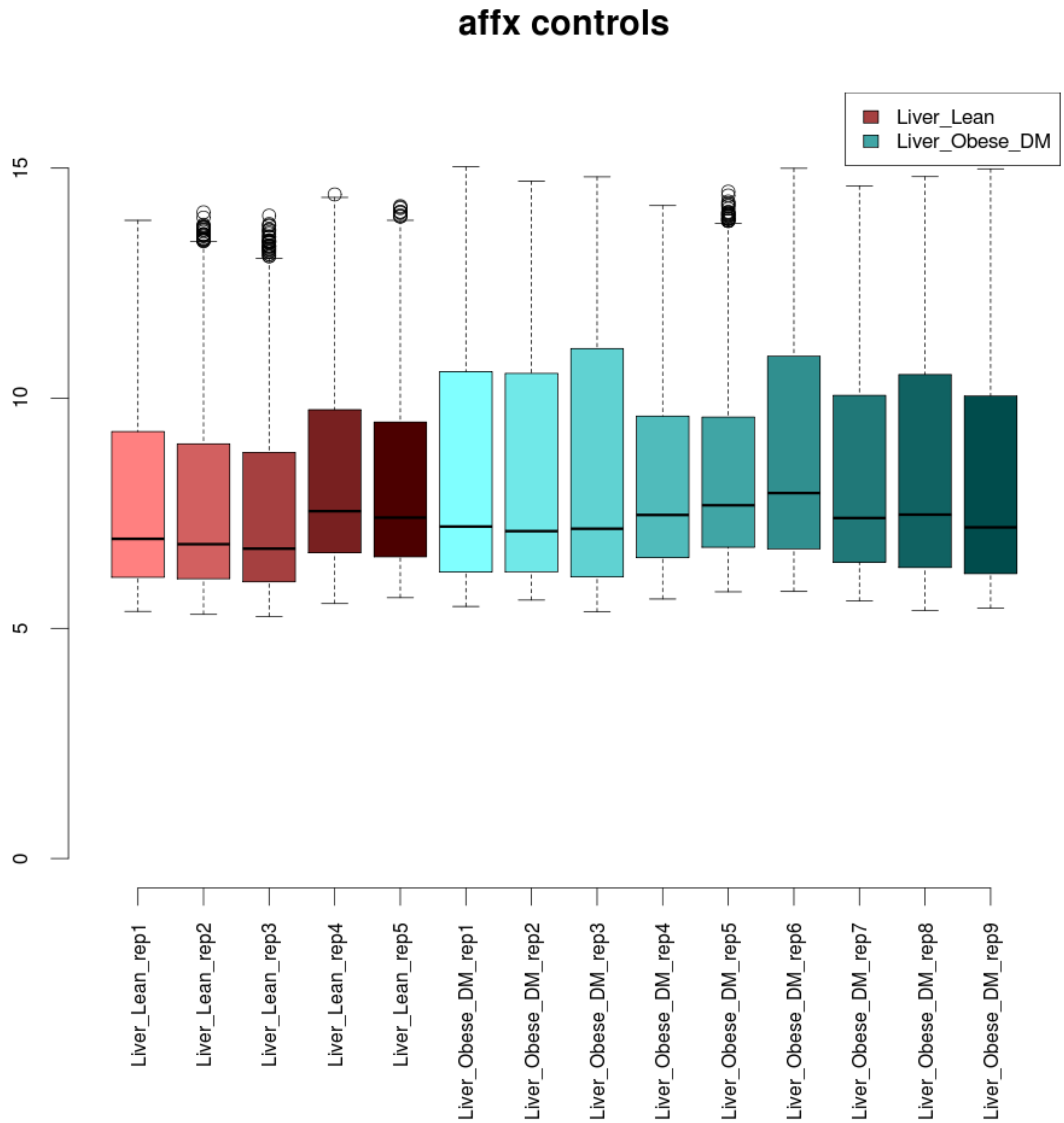

affx controls
Liver_Lean
Liver_Obese_DM
15
10
5
0
Liver_Lean_rep1
Liver_Lean_rep2
Liver_Lean_rep3
Liver_Lean_rep4
Liver_Lean_rep5
Liver_Obese_DM_rep1
Liver_Obese_DM_rep2
Liver_Obese_DM_rep3
Liver_Obese_DM_rep4
Liver_Obese_DM_rep5
Liver_Obese_DM_rep6
Liver_Obese_DM_rep7
Liver_Obese_DM_rep8
Liver_Obese_DM_rep9

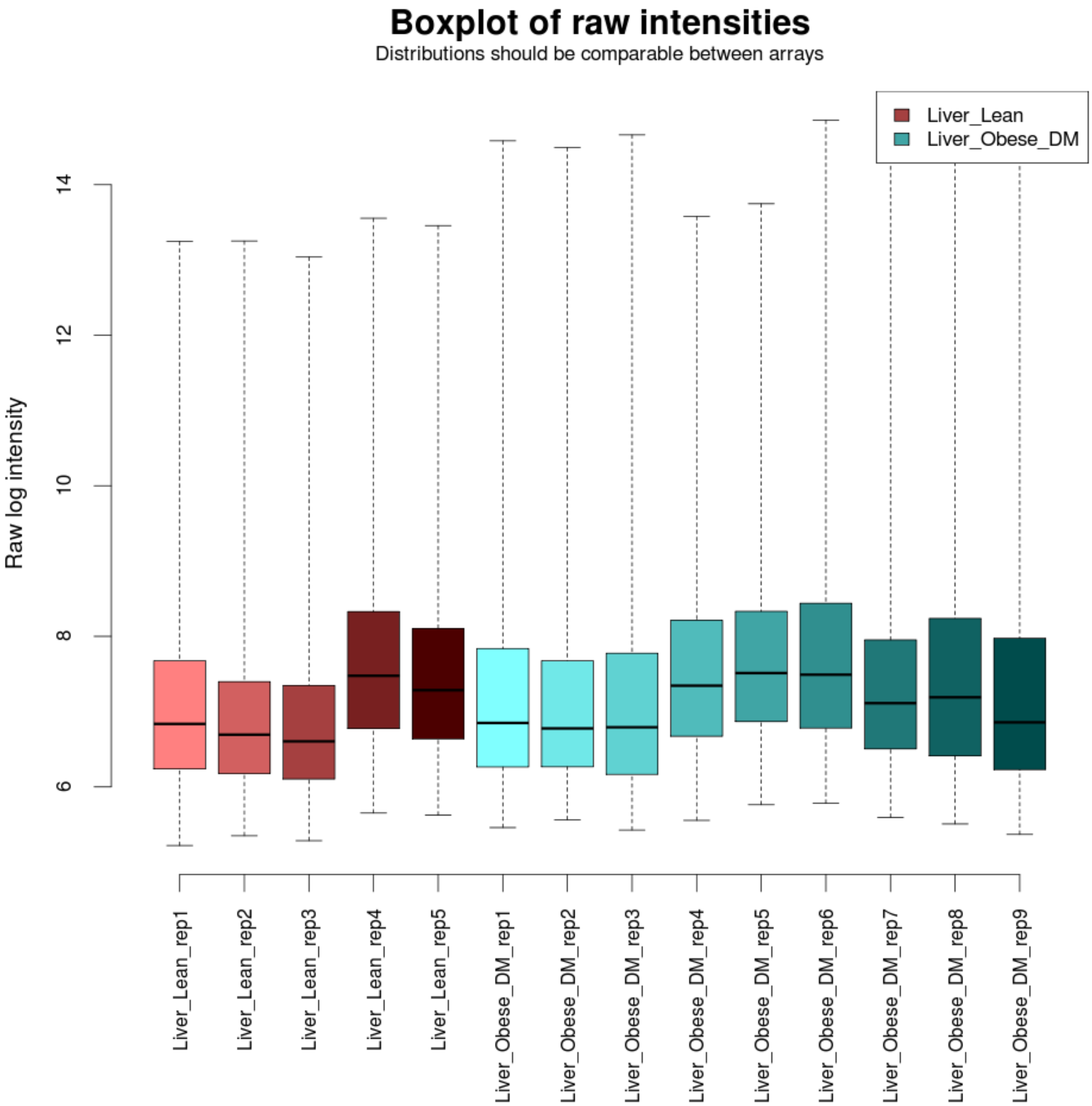

Boxplot of raw intensities
Distributions should be comparable between arrays
Liver_Lean
Liver_Obese_DM
Raw log intensity
6
8
10
12
14
Liver_Lean_rep1
Liver_Lean_rep2
Liver_Lean_rep3
Liver_Lean_rep4
Liver_Lean_rep5
Liver_Obese_DM_rep1
Liver_Obese_DM_rep2
Liver_Obese_DM_rep3
Liver_Obese_DM_rep4
Liver_Obese_DM_rep5
Liver_Obese_DM_rep6
Liver_Obese_DM_rep7
Liver_Obese_DM_rep8
Liver_Obese_DM_rep9

# MA plots of raw data

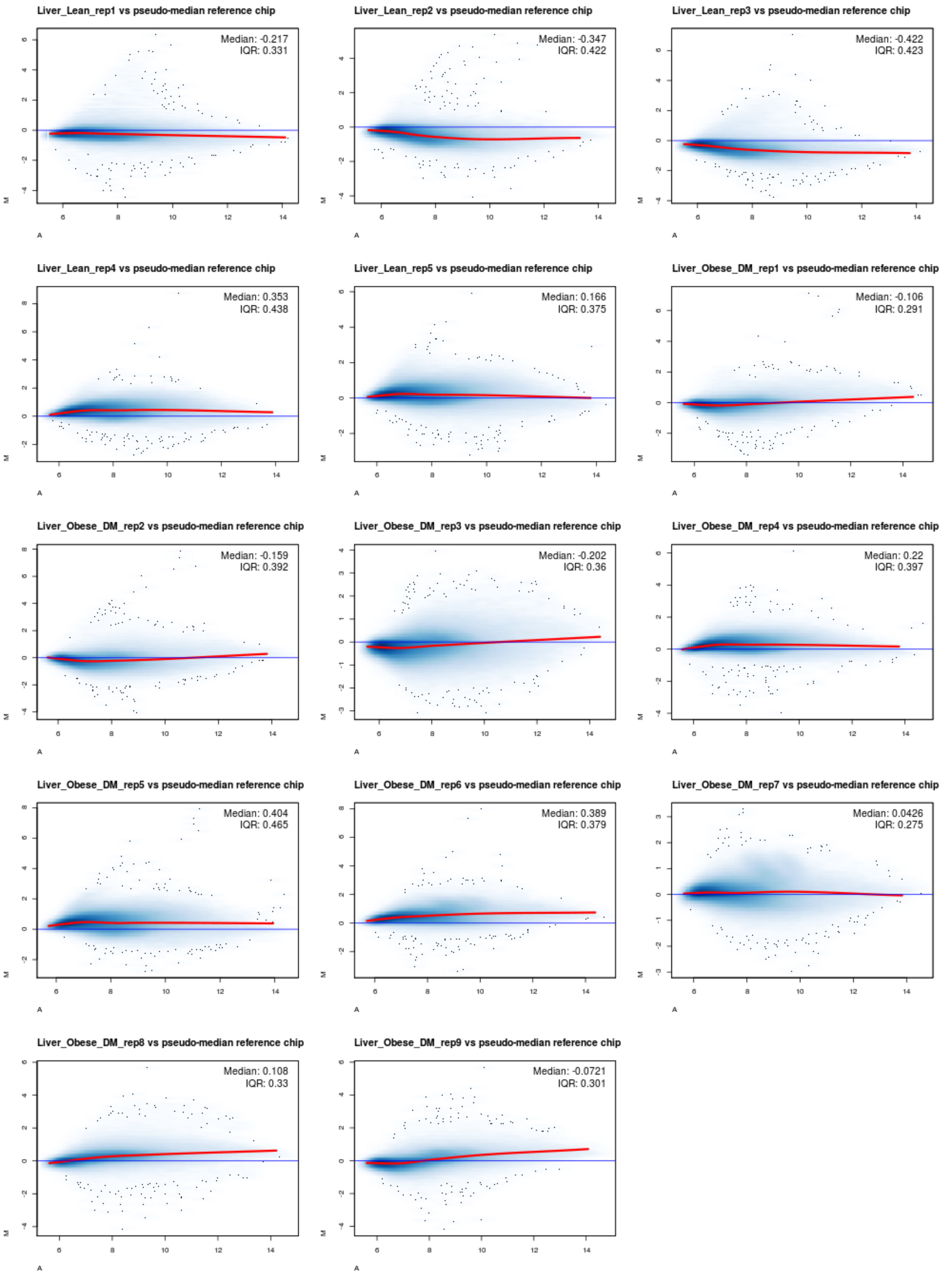

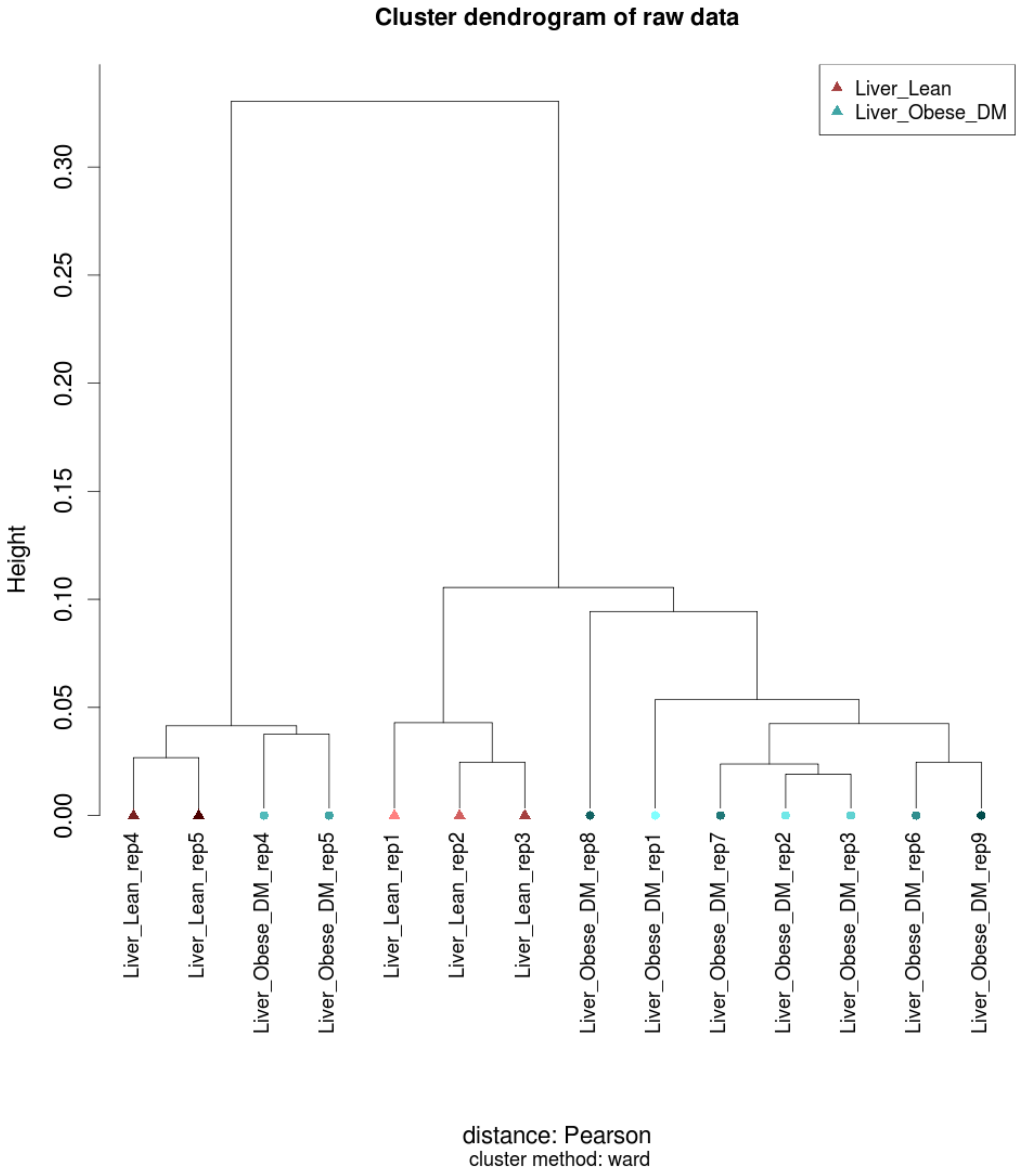
Cluster dendrogram of raw data
Liver_Lean
Liver_Obese_DM
Height
0.00
0.05
0.10
0.15
0.20
0.25
0.30
Liver_Lean_rep4
Liver_Lean_rep5
Liver_Obese_DM_rep4
Liver_Obese_DM_rep5
Liver_Lean_rep1
Liver_Lean_rep2
Liver_Lean_rep3
Liver_Obese_DM_rep8
Liver_Obese_DM_rep1
Liver_Obese_DM_rep7
Liver_Obese_DM_rep2
Liver_Obese_DM_rep3
Liver_Obese_DM_rep6
Liver_Obese_DM_rep9
distance: Pearson
cluster method: ward

# Pre-processing of Raw Data

Method: GCRMA
Annotation: hgu133a_Hs_ENSG

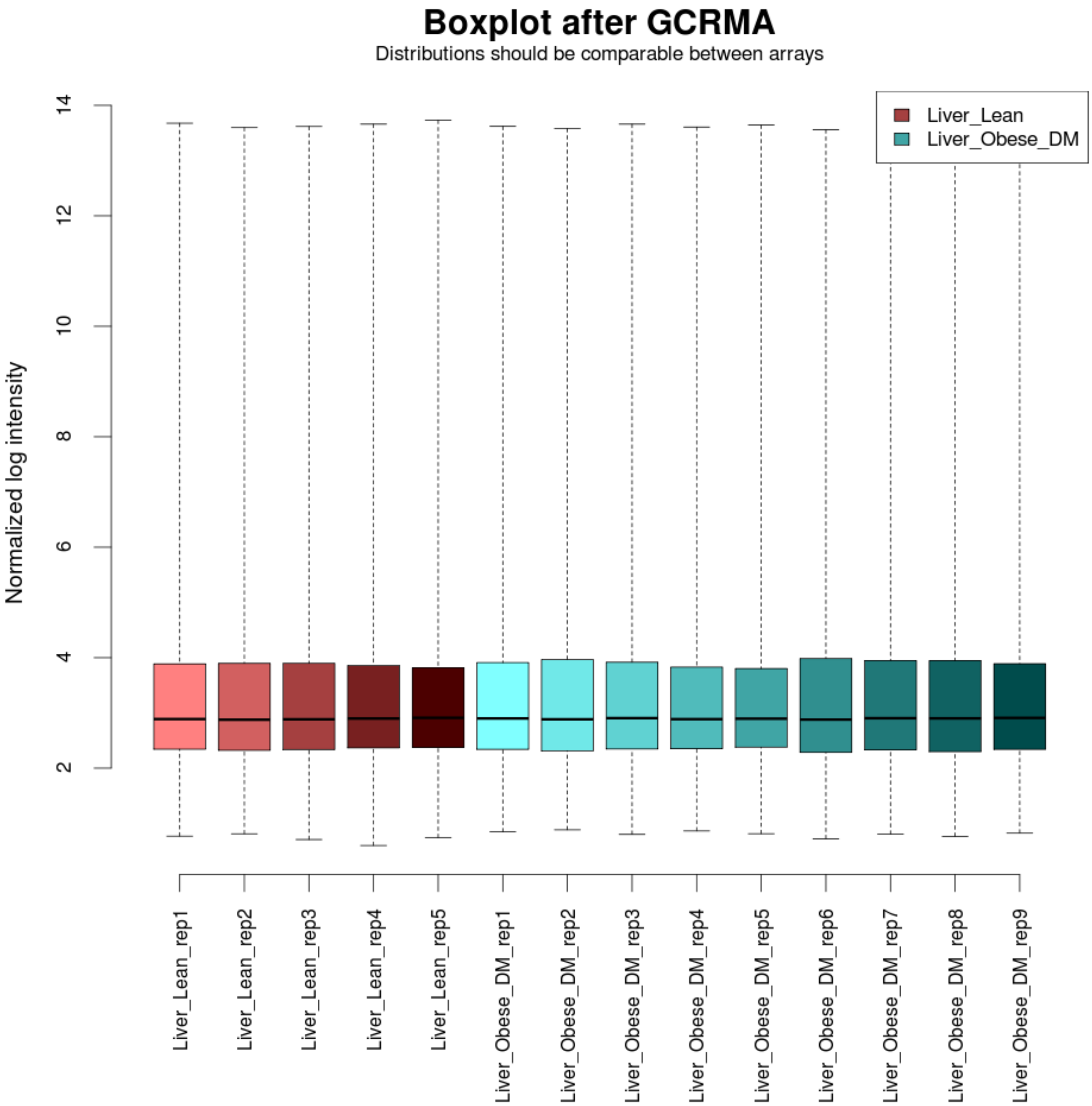

Boxplot after GCRMA
Distributions should be comparable between arrays
Normalized log intensity
2
4
6
8
10
12
14
Liver_Lean
Liver_Obese_DM
Liver_Lean_rep1
Liver_Lean_rep2
Liver_Lean_rep3
Liver_Lean_rep4
Liver_Lean_rep5
Liver_Obese_DM_rep1
Liver_Obese_DM_rep2
Liver_Obese_DM_rep3
Liver_Obese_DM_rep4
Liver_Obese_DM_rep5
Liver_Obese_DM_rep6
Liver_Obese_DM_rep7
Liver_Obese_DM_rep8
Liver_Obese_DM_rep9

# MA plots afterGCRMAnormalization

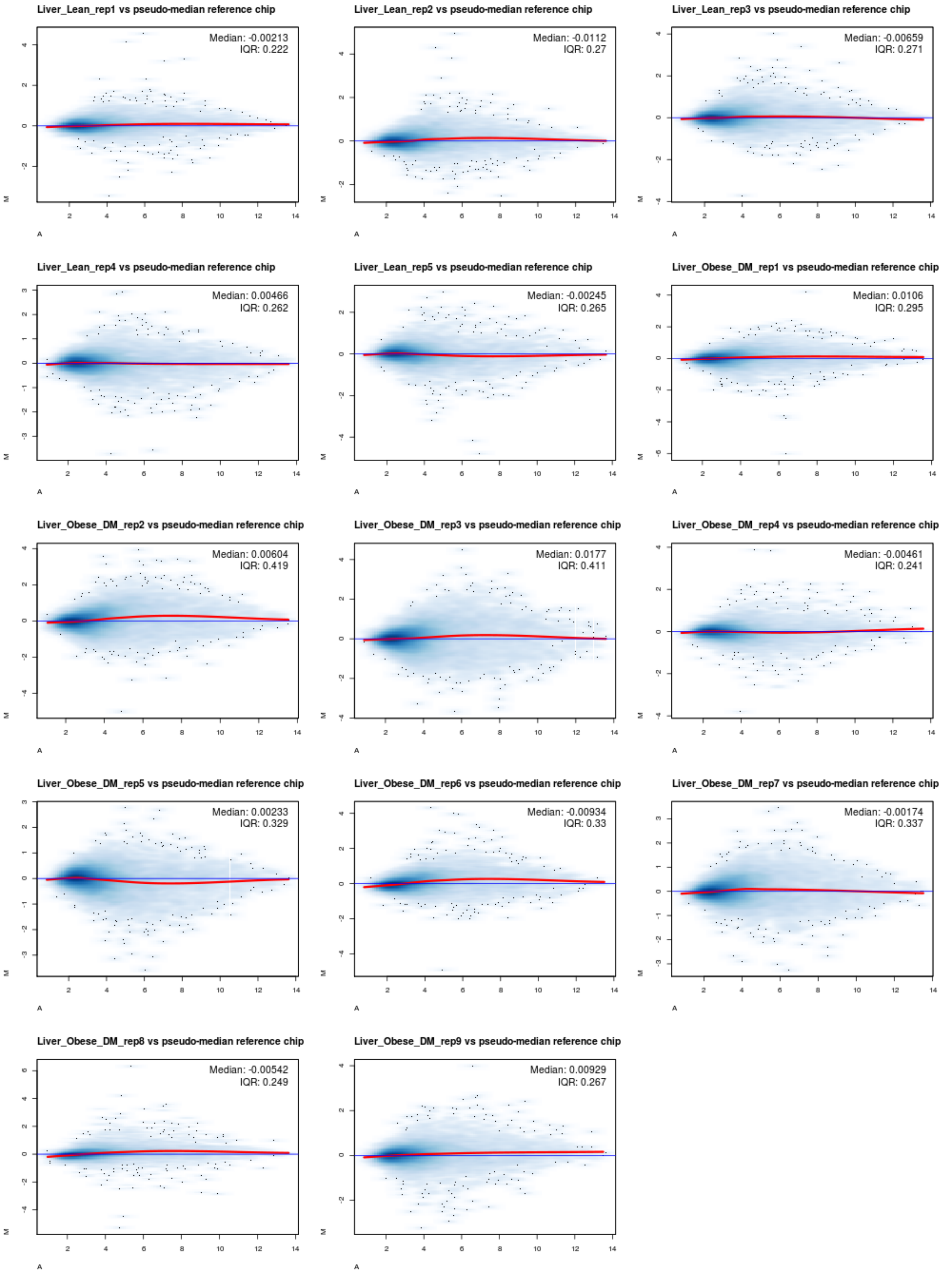

# PCA analysis after GCRMA normalization

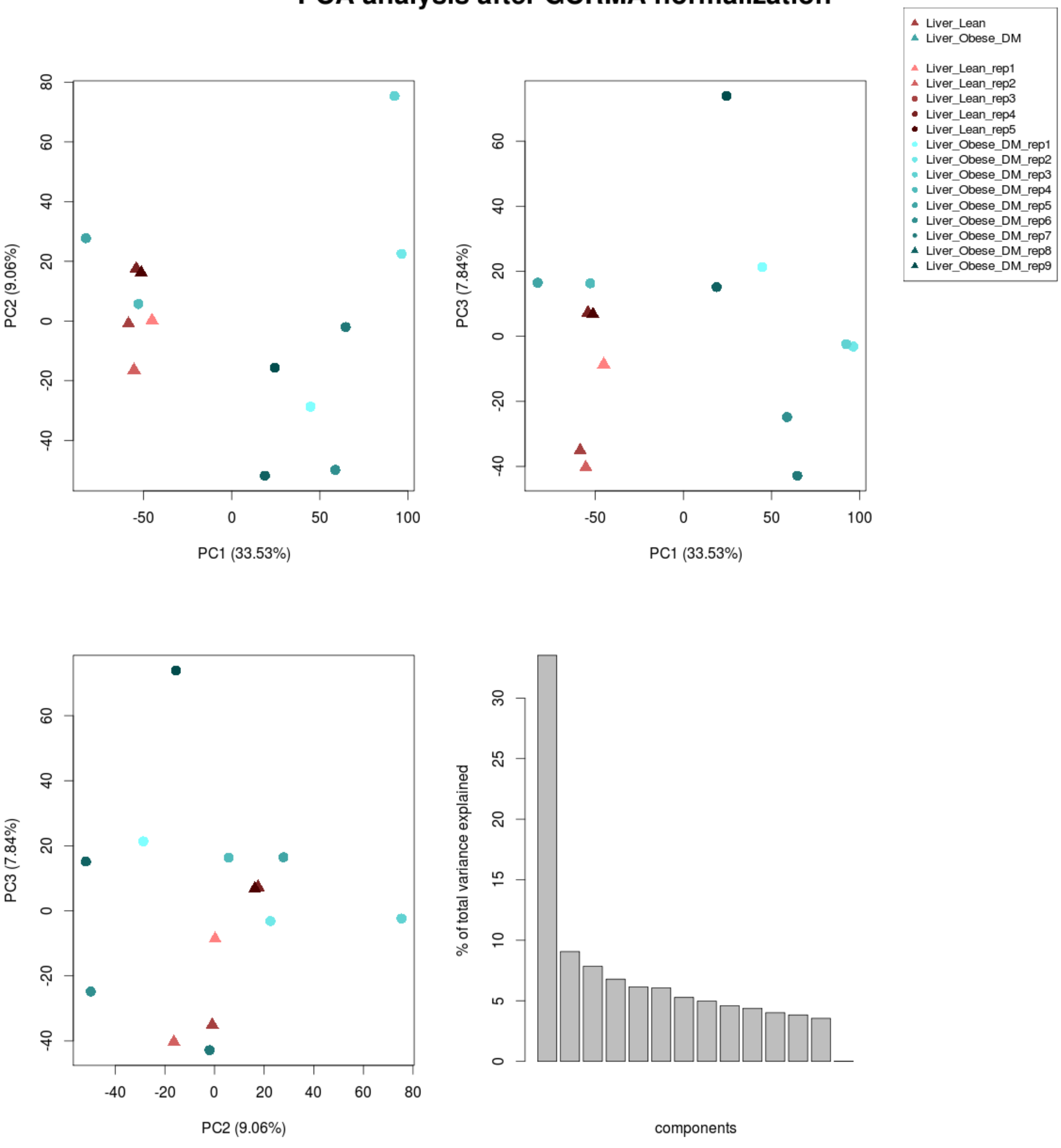

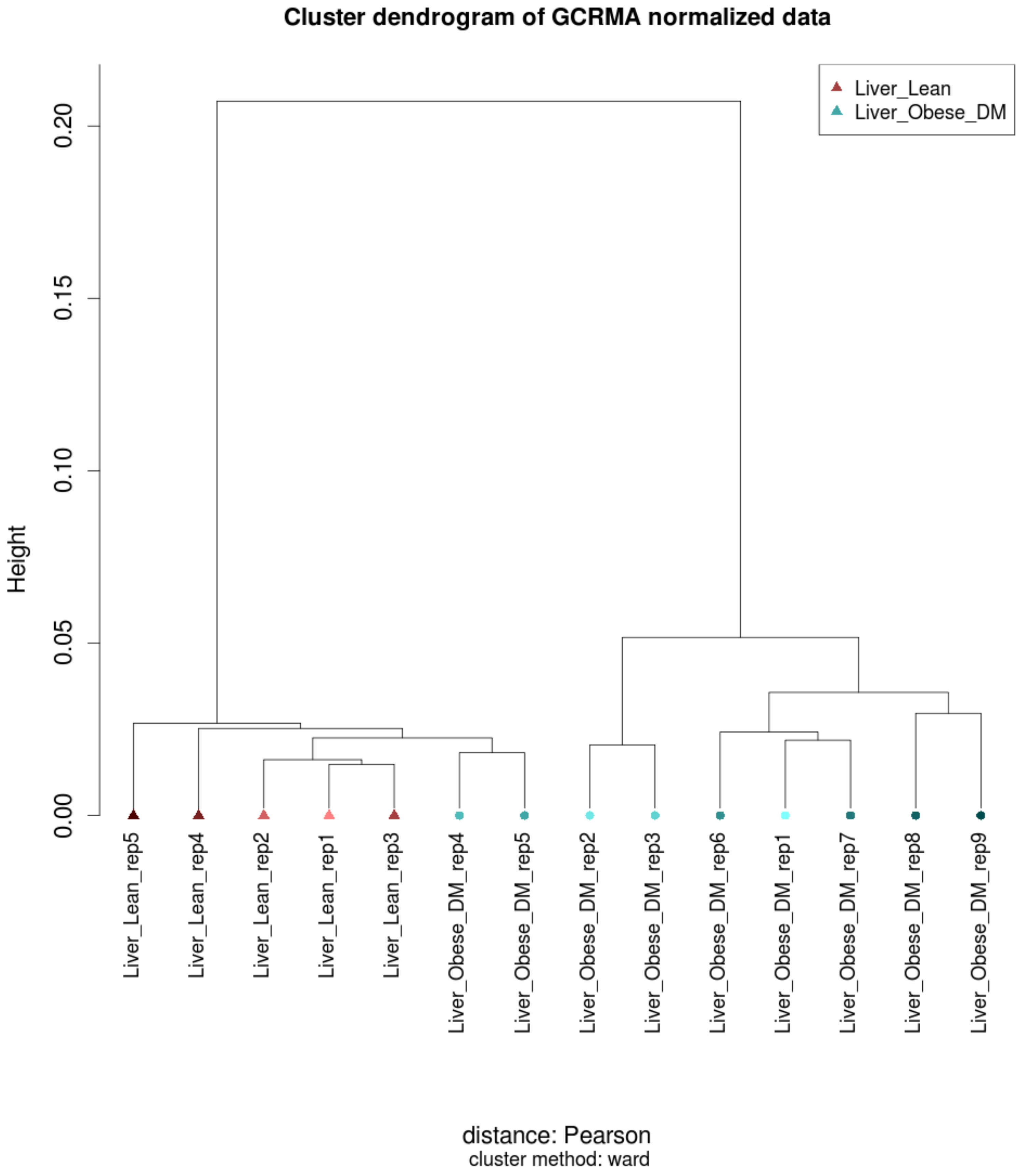
Cluster dendrogram of GCRMA normalized data
Liver_Lean
Liver_Obese_DM
Height
0.00
0.05
0.10
0.15
0.20
Liver_Lean_rep5
Liver_Lean_rep4
Liver_Lean_rep2
Liver_Lean_rep1
Liver_Lean_rep3
Liver_Obese_DM_rep4
Liver_Obese_DM_rep5
Liver_Obese_DM_rep2
Liver_Obese_DM_rep3
Liver_Obese_DM_rep6
Liver_Obese_DM_rep1
Liver_Obese_DM_rep7
Liver_Obese_DM_rep8
Liver_Obese_DM_rep9
distance: Pearson
cluster method: ward
