# Supplementary material for: A network biology workflow to study transcriptomics data of the diabetic liver
Source: BMC Genomics. 2014 Nov 15;15(1):971. doi: 10.1186/1471-2164-15-971 (PMC4246458; doi:10.1186/1471-2164-15-971)
Supplement: Supplementary file 3 — Additional file 3: Table S1. 118 upregulated genes in obese, diabetic patients with a fatty liver. Table S2. 63 downregulated genes in obese, diabetic patients with a fatty liver. (PDF 62 KB) [file 12864_2014_6667_MOESM3_ESM.pdf]

Table S1: 118 upregulated genes in obese. diabetic patients with a fatty liver

ENSG_ID	Gene Name	logFC	Fold Change	P.Value	Pathways
ENSG00000244734	HBB	2.57824443	5.97212528	0.000764109	WP176, WP1533, WP15
ENSG00000149925	ALDOA	2.197955637	4.588287004	0.017379001	WP706, WP534
ENSG00000188257	PLA2G2A	2.190144594	4.563512218	0.01326115	WP167, WP2431, WP2795, WP2533, WP1544
ENSG00000133048	CHI3L1	2.165028271	4.484752194	0.013082704	
ENSG00000167910	CYP7A1	2.09602294	4.275291944	0.032251198	WP43, WP702, WP430, WP2289, WP299
ENSG00000211899	IGHM	1.996139589	3.989310975	0.006051792	
ENSG00000108679	LGALS3BP	1.981884124	3.950086165	0.007849454	
ENSG00000130303	BST2	1.945892741	3.852761128	0.002626829	
ENSG00000099817	POLR2E	1.818837013	3.527966871	0.003655597	WP405
ENSG00000172354	GNB2	1.778945164	3.43175168	0.000901047	WP2355, WP536, WP289, WP35
ENSG00000159720	ATP6V0D1	1.737012906	3.333442648	0.007042804	WP2670
ENSG00000162496	DHRS3	1.707503887	3.265952677	0.007494973	WP716
ENSG00000135069	PSAT1	1.703645799	3.257230459	0.040292996	WP2005, WP2004, WP2002, WP2525
ENSG00000051523	CYBA	1.667030879	3.175603693	0.002789222	WP408, WP231
ENSG00000127948	POR	1.644338365	3.126044606	0.02979792	WP43
ENSG00000176974	SHMT1	1.643214589	3.123610548	0.006017827	WP1495, WP176, WP2525, WP241
ENSG00000161013	MGAT4B	1.642714986	3.122529034	0.001372285	
ENSG00000129255	MPDU1	1.633085905	3.101757513	0.017916463	WP2004, WP2003, WP2002
ENSG00000132693	CRP	1.607494709	3.047222212	0.022614098	WP364, WP176, WP1533, WP15
ENSG00000174903	RAB1B	1.605268298	3.042523273	0.001830172	
ENSG00000149809	TM7SF2	1.553035154	2.934338187	0.012289224	
ENSG00000100344	PNPLA3	1.546158078	2.920383995	0.009845551	WP167, WP236
ENSG00000111684	LPCAT3	1.520399713	2.868705189	0.00138912	
ENSG00000149923	PPP4C	1.518993336	2.865910062	0.00578602	
ENSG00000172270	BSG	1.518268618	2.864470773	0.005495582	WP129
ENSG00000169692	AGPAT2	1.46791775	2.766223548	0.011325478	WP325, WP236, WP2377
ENSG00000124145	SDC4	1.46417178	2.759050342	0.009155322	
ENSG00000130429	ARPC1B	1.460795312	2.752600639	0.007534227	WP2272
ENSG00000162511	LAPTM5	1.450917612	2.733818779	0.006470901	
ENSG00000167244	IGF2	1.448878946	2.72995836	0.00118409	WP474, WP254, WP2406
ENSG00000241685	ARPC1A	1.438281927	2.709979479	0.00703604	WP2272
ENSG00000110756	HPS5	1.425346763	2.685790478	0.002668343	
ENSG00000026025	VIM	1.416943871	2.67019272	0.008252439	WP383, WP69, WP2431, WP2572, WP2328
ENSG00000075624	ACTB	1.411733254	2.660566114	0.037191111	WP2118, WP306, WP289, WP51, WP2272
ENSG00000173369	C1QB	1.39178412	2.624029829	0.016328553	WP2431, WP558, WP545

ENSG00000148396	SEC16A	1.390998302	2.622600942	0.006109185	
ENSG00000019169	MARCO	1.383225571	2.60850929	0.007137312	
ENSG00000168056	LTBP3	1.323137524	2.502096659	0.005422454	
ENSG00000166741	NNMT	1.312364484	2.483482334	0.007378198	WP702, WP704
ENSG00000160932	LY6E	1.305018948	2.470869726	0.006993408	
ENSG00000165140	FBP1	1.300967458	2.463940568	0.001910973	WP534
ENSG00000145708	CRHBP	1.285898711	2.438338991	0.044361702	WP2355
ENSG00000197858	GPAA1	1.282570567	2.432720485	0.006159732	
ENSG00000234745	HLA-B	1.28076697	2.4296811	0.007462323	WP2328, WP619, WP183
ENSG00000108518	PFN1	1.268220032	2.408642088	0.007586814	WP524, WP51
ENSG00000058262	SEC61A1	1.265032693	2.40332656	0.039533576	
ENSG00000126247	CAPNS1	1.263359479	2.400540838	0.023856025	WP185, WP1772
ENSG00000163702	IL17RC	1.263152633	2.400196685	0.001496632	WP2112
ENSG00000179950	PUF60	1.257212048	2.390333716	0.021149059	
ENSG00000108639	SYNGR2	1.253977367	2.384980328	0.027688345	
ENSG00000183048	SLC25A10	1.239868532	2.361770093	0.006386273	
ENSG00000130005	GAMT	1.231223661	2.347660287	0.008677952	WP497
ENSG00000124762	CDKN1A	1.226748799	2.340389741	0.019668944	WP2100, WP707, WP1403, WP2586, WP236, WP1742, WP138, WP2572, WP2446, WP673, WP615, WP2261, WP2377, WP45, WP2005, WP2516, WP2004, WP179, WP710, WP1530, WP61, WP1545
ENSG00000115286	NDUFS7	1.224146016	2.336171222	0.025637594	WP623, WP111
ENSG00000105404	RABAC1	1.221829699	2.332423396	0.014988368	
ENSG00000177697	CD151	1.214326538	2.320324428	0.030108667	WP2572
ENSG00000099783	HNRNPM	1.208665785	2.31123793	0.007370349	WP2006, WP2004, WP2003, WP2002, WP411
ENSG00000160888	IER2	1.20792067	2.310044542	0.03510725	
ENSG00000137204	SLC22A7	1.206176	2.307252664	0.01758174	WP1601
ENSG00000125968	ID1	1.201625453	2.299986595	0.047524279	WP2064, WP53
ENSG00000108179	PPIF	1.199937771	2.297297617	0.0215857	WP2005, WP2004, WP2003, WP2002
ENSG00000172531	PPP1CA	1.192152475	2.284933971	0.023571048	WP1602
ENSG00000100412	ACO2	1.191655612	2.284147177	0.012242033	WP78
ENSG00000206066	IGLL3P	1.190764377	2.282736563	0.033924688	
ENSG00000119431	HDHD3	1.188885892	2.279766224	0.018662299	
ENSG00000165215	CLDN3	1.187102275	2.276949469	0.001796385	
ENSG00000078902	TOLLIP	1.177926575	2.262513774	0.005315084	WP2637, WP1449, WP195, WP75, WP1772
ENSG00000168961	LGALS9	1.175467845	2.258661142	6.50E-05	
ENSG00000171298	GAA	1.173979296	2.256331895	0.015207212	
ENSG00000185000	DGAT1	1.164115668	2.240958089	0.017344365	WP325, WP430
ENSG00000163684	RPP14	1.163386592	2.239825893	0.006685674	
ENSG00000177106	EPS8L2	1.154830944	2.226582308	0.007092125	

ENSG00000030582	GRN	1.154312279	2.225781971	0.013421299	WP1984					
ENSG00000167797	CDK2AP2	1.149162156	2.217850555	0.006763358						
ENSG00000100292	HMOX1	1.143993652	2.209919246	0.000701922	WP2006, WP408, WP2005, WP2004, WP2003, WP2002, WP3					
ENSG00000129988	LBP	1.13923878	2.202647726	0.005514519	WP1449, WP75					
ENSG00000143761	ARF1	1.136921072	2.199111985	0.011838157	WP1846, WP179, WP481, WP2377					
ENSG00000164889	SLC4A2	1.135517633	2.196973752	0.006233982						
ENSG00000155666	KDM8	1.13309295	2.193284481	0.007765253						
ENSG00000131446	MGAT1	1.13049673	2.189341079	0.005585514						
ENSG00000149150	SLC43A1	1.120076116	2.1735844	0.008612709						
ENSG00000169564	PCBP1	1.116795257	2.168647028	0.006298712						
ENSG00000109758	HGFAC	1.106337872	2.15298441	0.007169787						
ENSG00000204622	HLA-J	1.104366723	2.150044802	0.006827329	WP183					
ENSG00000196365	LONP1	1.102644235	2.147479314	0.020104043						
ENSG00000182087	TMEM259	1.100907253	2.144895339	0.006397311						
ENSG00000169136	ATF5	1.096832715	2.138846156	0.02123705	WP289					
ENSG00000126457	PRMT1	1.072657113	2.103303604	0.020681541	WP585, WP411, WP465					
ENSG00000157399	ARSE	1.066241373	2.093970871	0.032533792	WP697					
ENSG00000110244	APOA4	1.06484564	2.091946041	0.027701154	WP430					
ENSG00000025434	NR1H3	1.061183473	2.086642536	0.008875053	WP236, WP2011, WP170, WP299					
ENSG00000101150	TPD52L2	1.057773877	2.081716889	0.003458577						
ENSG00000160221	C21orf33	1.057418812	2.081204616	0.005674083	WP2361					
ENSG00000211666	IGLV2-14	1.054317602	2.076735674	0.020049833						
ENSG00000164050	PLXNB1	1.049979753	2.070500789	0.001019	WP1907					
ENSG00000167671	UBXN6	1.046090432	2.064926502	0.004438548						
ENSG00000138030	KHK	1.043751741	2.06158185	0.010641968	WP690					
ENSG00000155366	RHOC	1.043655427	2.061444224	0.011930525	WP2431, WP1907					
ENSG00000214063	TSPAN4	1.04209551	2.059216487	0.006241956						
ENSG00000204592	HLA-E	1.041266891	2.058034106	0.004601341	WP2328, WP183					
ENSG00000128294	TPST2	1.039953857	2.056161888	0.010798857						
ENSG00000141505	ASGR1	1.038397219	2.053944529	0.024549974						
ENSG00000099834	CDHR5	1.037443286	2.052586876	0.036719037						
ENSG00000105401	CDC37	1.033378631	2.046812048	0.003558305	WP2100, WP2586, WP231					
ENSG00000115425	PECR	1.030682524	2.04299054	0.044027101	WP368, WP357, WP2328					
ENSG00000101940	WDR13	1.030294869	2.042441659	0.024935436						
ENSG00000072310	SREBF1	1.027026864	2.037820339	0.034389181	WP1982, WP1403, WP197, WP236, WP2011, WP53					
ENSG00000211653	IGLV1-40	1.026038251	2.036424391	0.00020154						
ENSG00000167588	GPD1	1.020050589	2.027990071	0.008308088	WP325, WP2533					
ENSG00000143641	GALNT2	1.01660679	2.023154912	0.006028017	WP1424					

ENSG00000013583	HEBP1	1.016019478	2.022331465	0.006733645	
ENSG00000163902	RPN1	1.015421149	2.021492917	0.031560568	WP183
ENSG00000095321	CRAT	1.011858446	2.016507045	0.008654991	WP143
ENSG00000122971	ACADS	1.011139862	2.015502904	0.028124311	WP143, WP368
ENSG00000108294	PSMB3	1.010439227	2.014524326	0.019785222	WP183
ENSG00000182054	IDH2	1.005791903	2.008045422	0.007463141	WP78
ENSG00000137491	SLCO2B1	1.003658478	2.005078163	0.016444327	
ENSG00000173372	C1QA	1.002180247	2.003024749	0.038943621	WP558, WP545

Table S2: 63 downregulated genes in obese. diabetic patients with a fatty liver

ENSG_ID	Gene name	logFC	Fold Change	P.Value	Pathways
ENSG00000165841	CYP2C19	-2.499888696	-5.656417841	0.013964359	WP691, WP43, WP702, WP2491
ENSG00000229807	XIST	-2.341783511	-5.069289339	0.034413414	
ENSG00000213002	AC120194.1	-2.279212072	-4.854127731	0.009482099	
ENSG00000181656	GPR88	-2.070691183	-4.200878857	0.00414156	WP117
ENSG00000114698	PLSCR4	-1.769003687	-3.40818509	0.014828106	
ENSG00000148935	GAS2	-1.642620824	-3.122325238	0.003076903	
ENSG00000081154	PCNP	-1.615206055	-3.063553519	0.01438264	
ENSG00000186106	ANKRD46	-1.607777839	-3.047820291	0.000458479	
ENSG00000066557	LRRC40	-1.565538338	-2.959879283	0.003290914	
ENSG00000118762	PKD2	-1.531741618	-2.891346712	0.00015577	
ENSG00000112893	MAN2A1	-1.509402559	-2.846921195	0.001760255	
ENSG00000134884	ARGLU1	-1.458064997	-2.747396241	0.008371124	
ENSG00000108176	DNAJC12	-1.422229966	-2.679994363	0.019984058	
ENSG00000118777	ABCG2	-1.415104936	-2.666791319	0.005212495	WP2670, WP2005, WP2004, WP1601, WP229
ENSG00000127324	TSPAN8	-1.399681041	-2.638432437	0.036283212	
ENSG00000123636	BAZ2B	-1.378150626	-2.599349498	0.003856292	
ENSG00000116473	RAP1A	-1.375872274	-2.595247759	0.007776669	WP382, WP2380, WP585, WP313, WP1984, WP185, WP306, WP437, WP734, WP2377, WP2032
ENSG00000112414	GPR126	-1.344124443	-2.538760749	0.011823822	
ENSG00000163694	RBM47	-1.341816848	-2.534703239	0.000323234	
ENSG00000146039	SLC17A4	-1.339818159	-2.53119413	1.32E-06	
ENSG00000096092	TMEM14A	-1.305352008	-2.471440215	0.012191165	
ENSG00000170525	PFKFB3	-1.29653479	-2.456381751	0.006943427	WP1403

ENSG00000083896	YTHDC1	-1.266666389	-2.406049609	0.016762605							
ENSG00000170142	UBE2E1	-1.252765335	-2.382977508	0.007435351							
ENSG00000133731	IMPA1	-1.249044948	-2.37684026	0.011640006	WP1984						
ENSG00000267458	CTC-425F1.4	-1.241202586	-2.363955022	0.008529498							
ENSG00000148468	FAM171A1	-1.22804966	-2.342500994	0.020196287							
ENSG00000188641	DPYD	-1.226849491	-2.340553092	0.032183328	WP1601						
ENSG00000164128	NPY1R	-1.223783928	-2.335584963	0.026114846	WP455, WP2197, WP24						
ENSG00000168679	SLC16A4	-1.220904389	-2.330927914	0.007796172							
ENSG00000069956	MAPK6	-1.211857885	-2.316357425	0.000483686	WP382, WP185, WP26, WP306, WP51, WP481						
ENSG00000172869	DMXL1	-1.201050407	-2.299070022	0.001972952							
ENSG00000067900	ROCK1	-1.162381563	-2.238266099	0.019484335	WP524, WP2324, WP2038, WP138, WP185, WP306, WP51, WP437, WP2272, WP1907, WP1544						
ENSG00000106028	SSBP1	-1.151824825	-2.221947648	0.006775775							
ENSG00000122873	CISD1	-1.131129488	-2.190301521	7.05E-05	WP236						
ENSG00000256612	CYP2B7P	-1.130053099	-2.188667956	0.047836821							
ENSG00000181163	NPM1	-1.12777282	-2.185211348	0.001100048							
ENSG00000125629	INSIG2	-1.108489953	-2.156198431	0.013767708	WP1982						
ENSG00000109466	KLHL2	-1.104400324	-2.150094879	0.003294354							
ENSG00000137960	GIPC2	-1.098863604	-2.14185914	0.002487617							
ENSG00000166922	SCG5	-1.094563965	-2.135485297	0.000966852							
ENSG00000143815	LBR	-1.092063316	-2.131787029	0.008952693	WP2363						
ENSG00000095564	BTAF1	-1.081056201	-2.115584339	0.000560757							
ENSG00000103121	CMC2	-1.077092717	-2.109780219	0.001235663							
ENSG00000109511	ANXA10	-1.068047602	-2.096594127	0.002498809							
ENSG00000084073	ZMPSTE24	-1.063523663	-2.090030018	0.00035131	WP236						
ENSG00000164172	MOCS2	-1.061306596	-2.086820624	0.037859551							
ENSG00000091428	RAPGEF4	-1.060792301	-2.086076841	0.007321893							
ENSG00000141198	TOM1L1	-1.059733927	-2.084547037	0.001920534							
ENSG00000180957	PITPNB	-1.058249638	-2.082403495	0.000121671							
ENSG00000072080	SPP2	-1.049640116	-2.070013413	0.011821139							
ENSG00000083223	ZCCHC6	-1.04640476	-2.065376448	0.002286542							
ENSG00000172007	RAB33B	-1.042520388	-2.059823023	0.007318608							
ENSG00000054277	OPN3	-1.042375407	-2.059616035	0.001620072	WP455						
ENSG00000179941	BBS10	-1.041849241	-2.058865009	0.00600885							
ENSG00000137628	DDX60	-1.040162158	-2.056458785	0.000598058							
ENSG00000115392	FANCL	-1.034560622	-2.048489676	0.007011127							
ENSG00000214413	BBIP1	-1.032170376	-2.045098564	0.002195937							
ENSG00000064726	BTBD1	-1.026425617	-2.036971249	0.0009802							
ENSG00000116678	LEPR	-1.020152525	-2.028133367	0.030809035	WP2034, WP1403						

ENSG00000187164	KIAA1598	-1.020007792	-2.027929913	0.047692094						
ENSG00000076258	FMO4	-1.014302925	-2.019926678	0.006610823	WP702, WP688					
ENSG00000181061	HIGD1A	-1.010534588	-2.014657489	0.005438154						
