# Supplementary material for: A network biology workflow to study transcriptomics data of the diabetic liver
Source: BMC Genomics. 2014 Nov 15;15(1):971. doi: 10.1186/1471-2164-15-971 (PMC4246458; doi:10.1186/1471-2164-15-971)
Supplement: Supplementary file 4 — Additional file 4: Figure S1–S7. Images of all seven altered pathways with dataset visualized on the pathways. (PDF 452 KB) [file 12864_2014_6667_MOESM4_ESM.pdf]

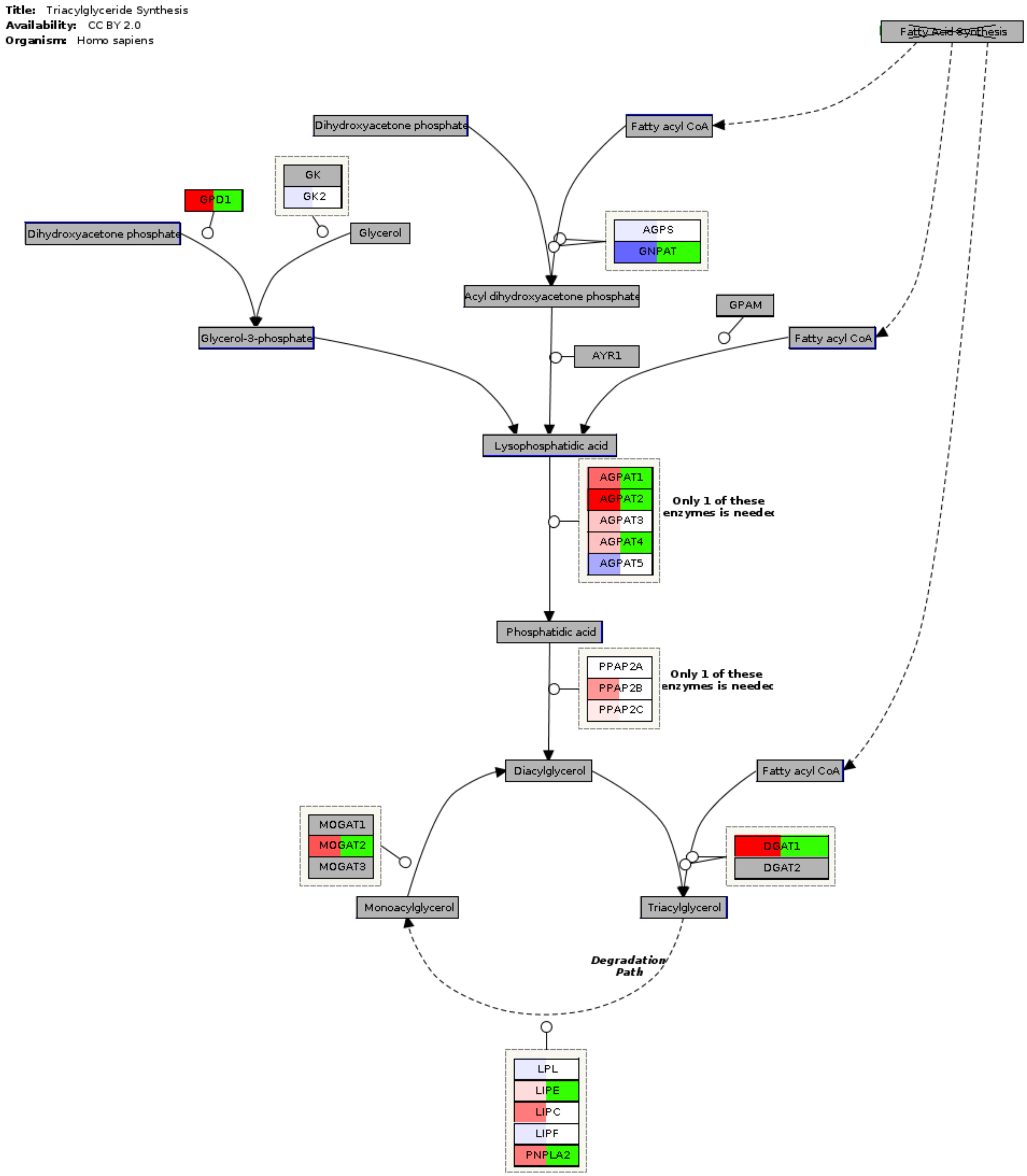

Figure S1: Triacylglyceride Synthesis pathway from WikiPathways. Diabetic, fatty liver dataset is visualized on gene products in the pathway. (http://www.wikipathways.org/instance/WP325)

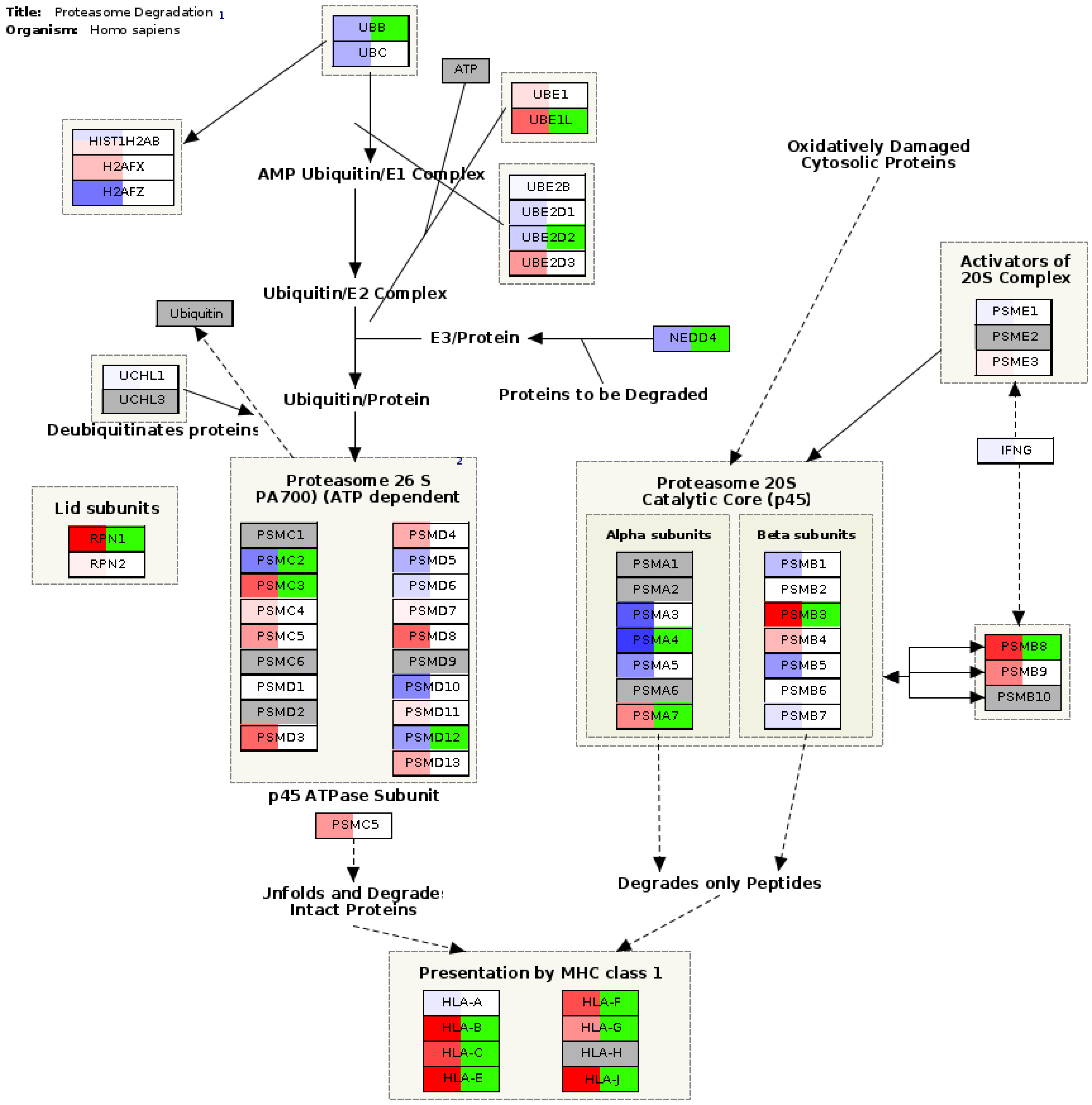

Figure S2: Proteasome Degradation pathway from WikiPathways. Diabetic, fatty liver dataset is visualized on gene products in the pathway. (http://www.wikipathways.org/instance/WP183)

Title: Statin Pathway
Availability: CC BY 2.0
Last modified: 10/16/2013
Organism: Homo sapiens

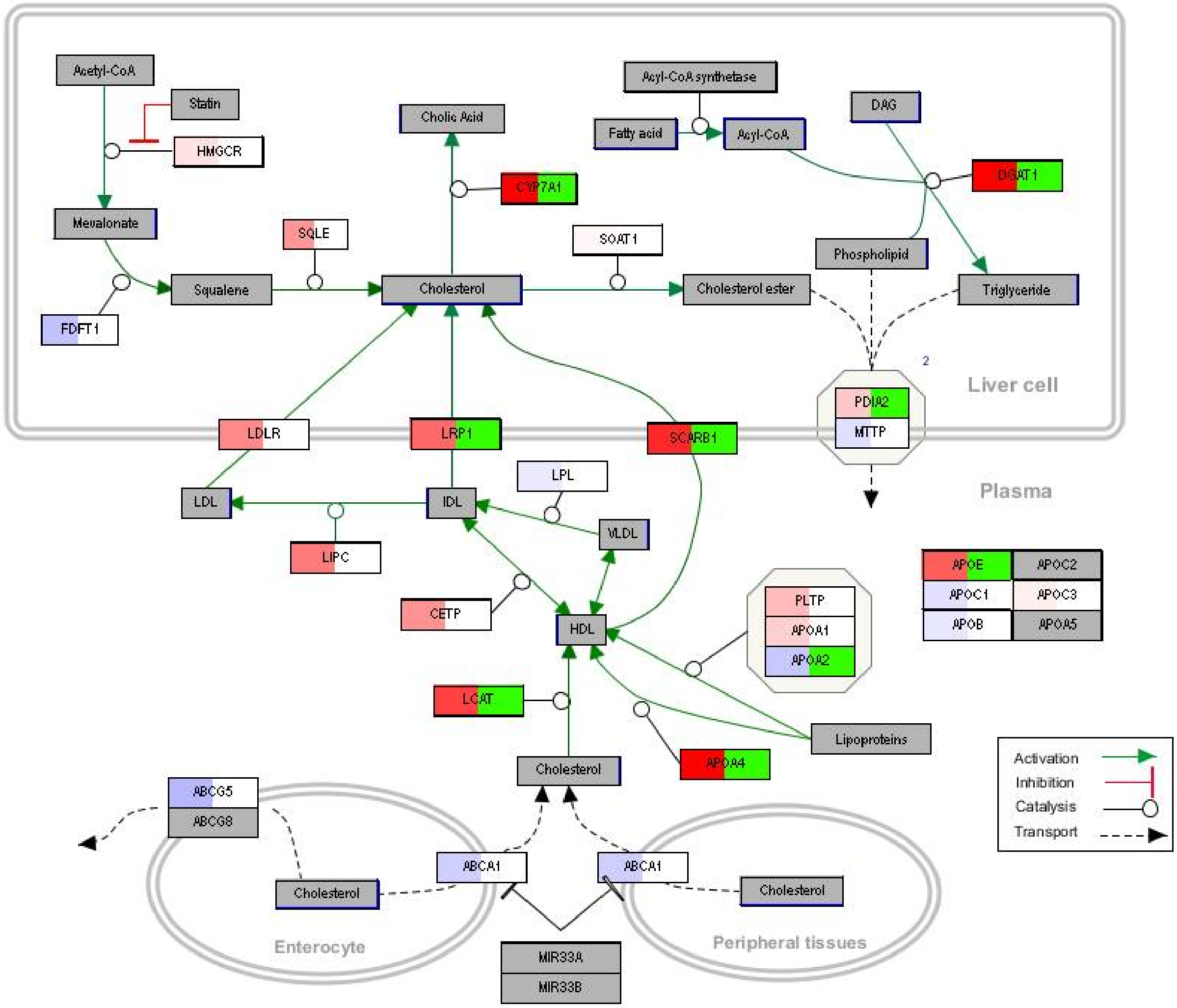

Figure S3: Statin pathway from WikiPathways. Diabetic, fatty liver dataset is visualized on gene products in the pathway. (http://www.wikipathways.org/instance/WP430)

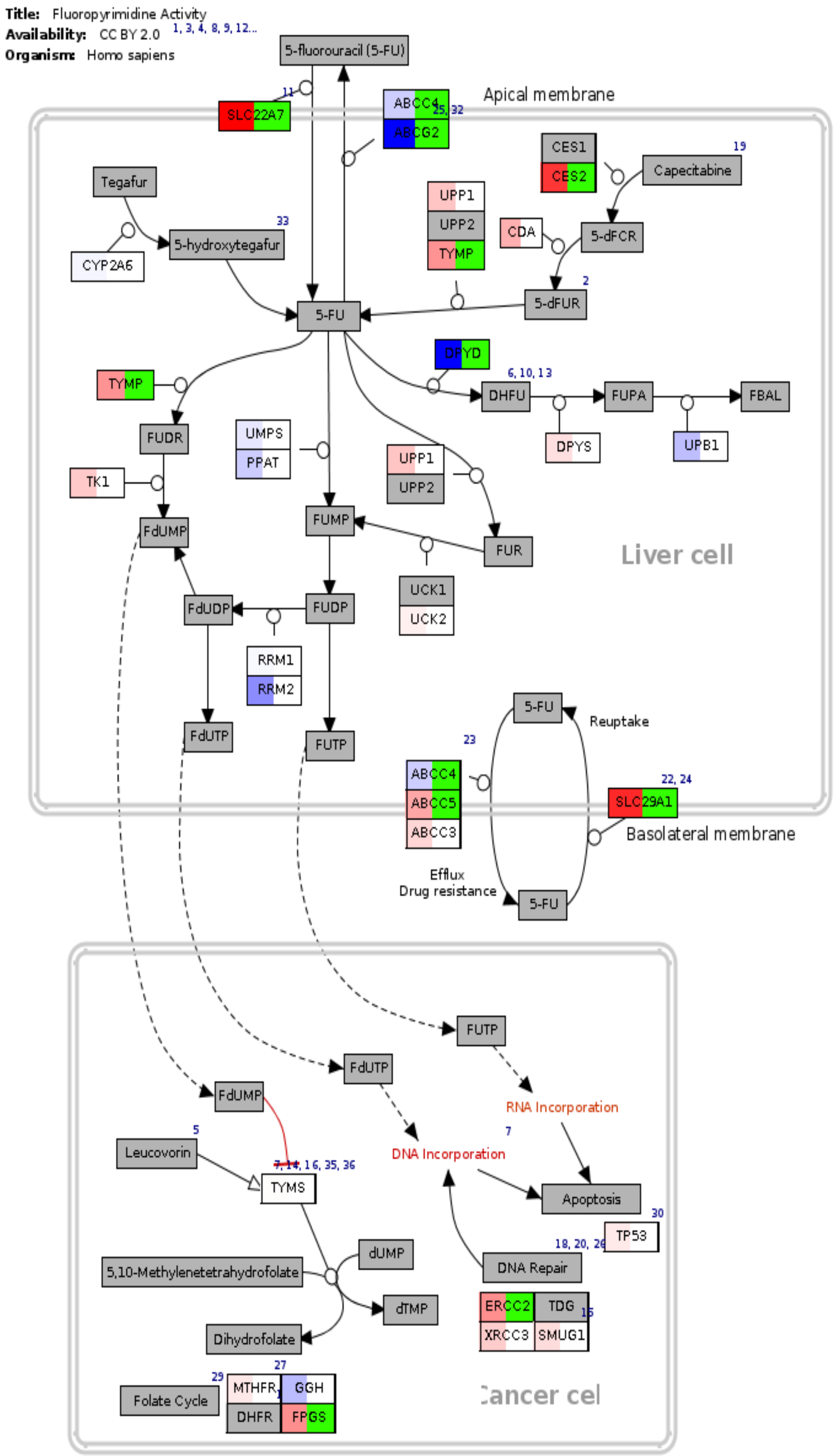

Figure S4: Fluoropyrimidine Activity pathway from WikiPathways. Diabetic, fatty liver dataset is visualized on gene products in the pathway. (http://www.wikipathways.org/instance/WP1601)

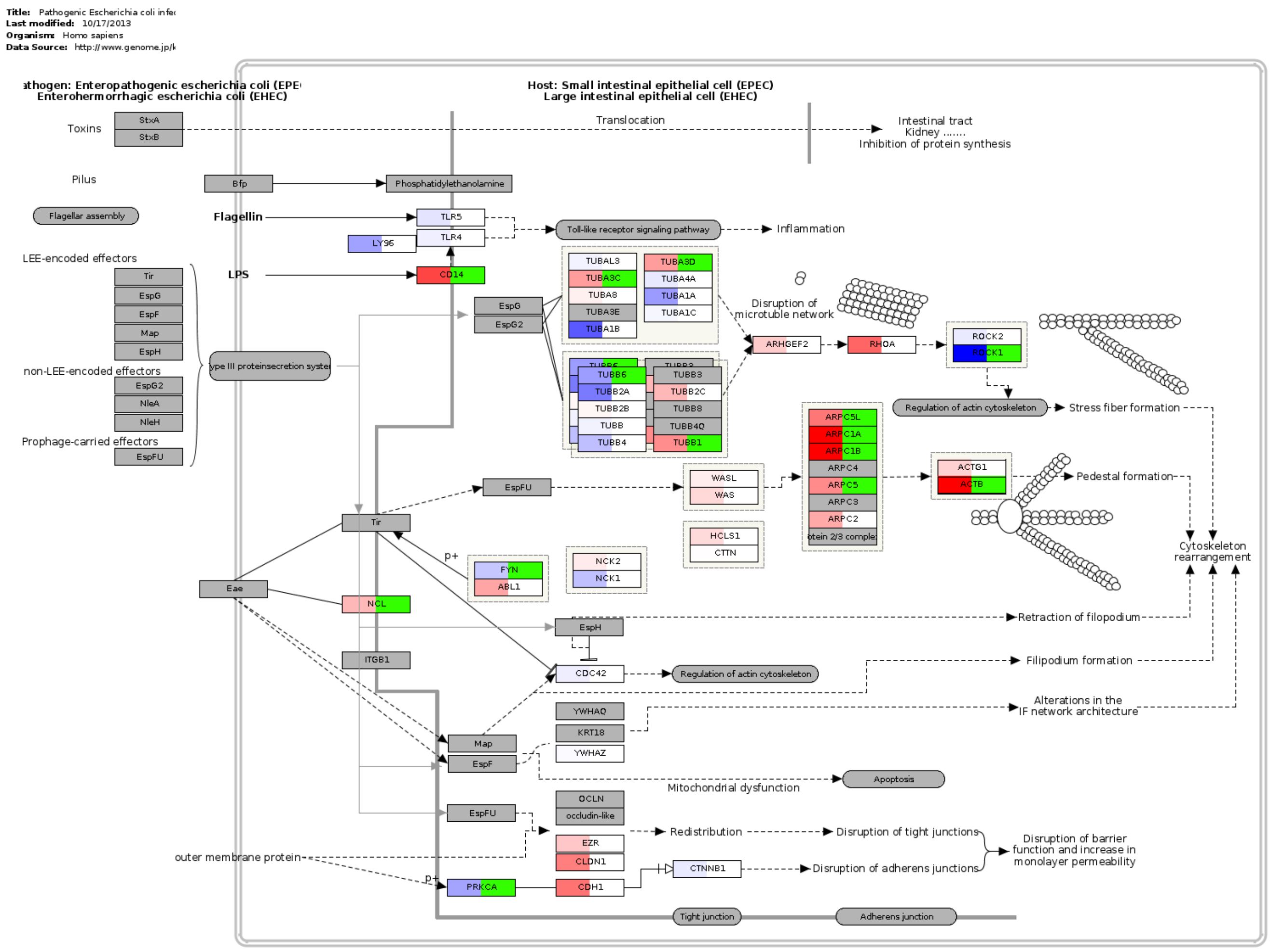

Figure S5: Pathogenic Escherichia coli infection pathway from WikiPathways. Diabetic, fatty liver dataset is visualized on gene products in the pathway. (http://www.wikipathways.org/instance/WP2272)

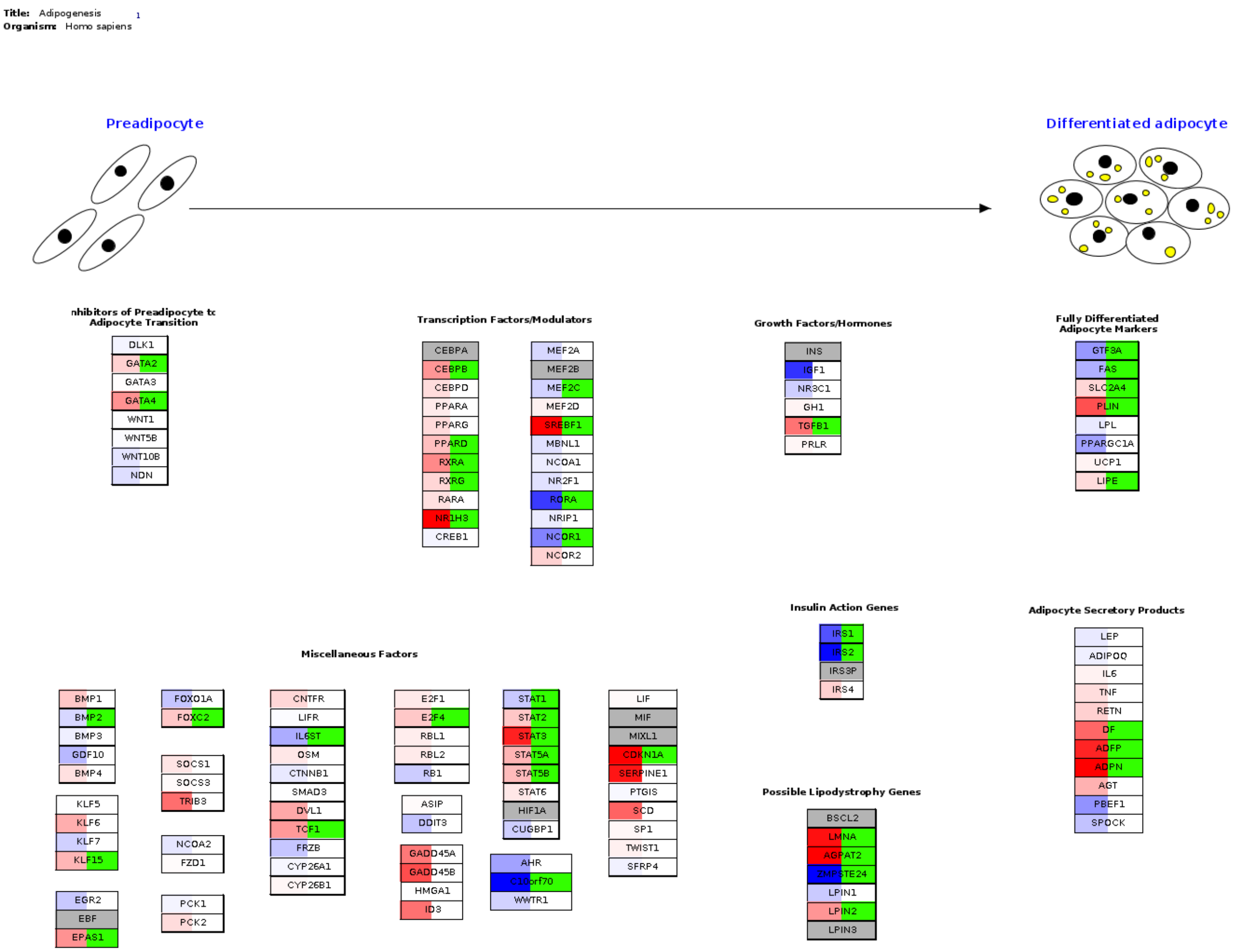

Figure S6: Adipogenesis pathway from WikiPathways. Diabetic, fatty liver dataset is visualized on gene products in the pathway. (http://www.wikipathways.org/instance/WP236)

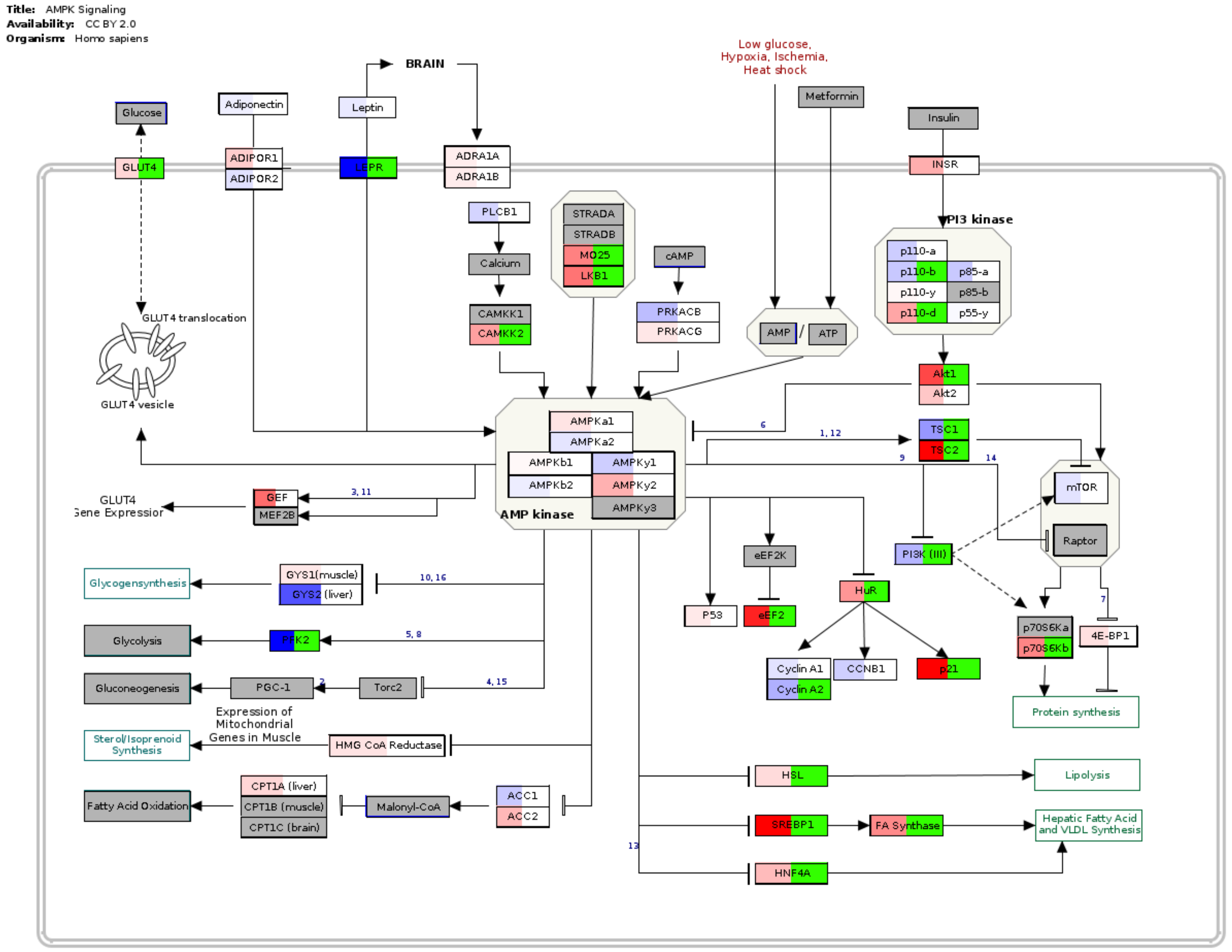

Figure S7: **AMPK Signaling pathway from WikiPathways.** Diabetic, fatty liver dataset is visualized on gene products in the pathway. (http://www.wikipathways.org/instance/WP1403)
