# Supplementary figures and images for: A network biology workflow to study transcriptomics data of the diabetic liver
Source: BMC Genomics. 2014 Nov 15;15(1):971. doi: 10.1186/1471-2164-15-971 (PMC4246458; doi:10.1186/1471-2164-15-971)

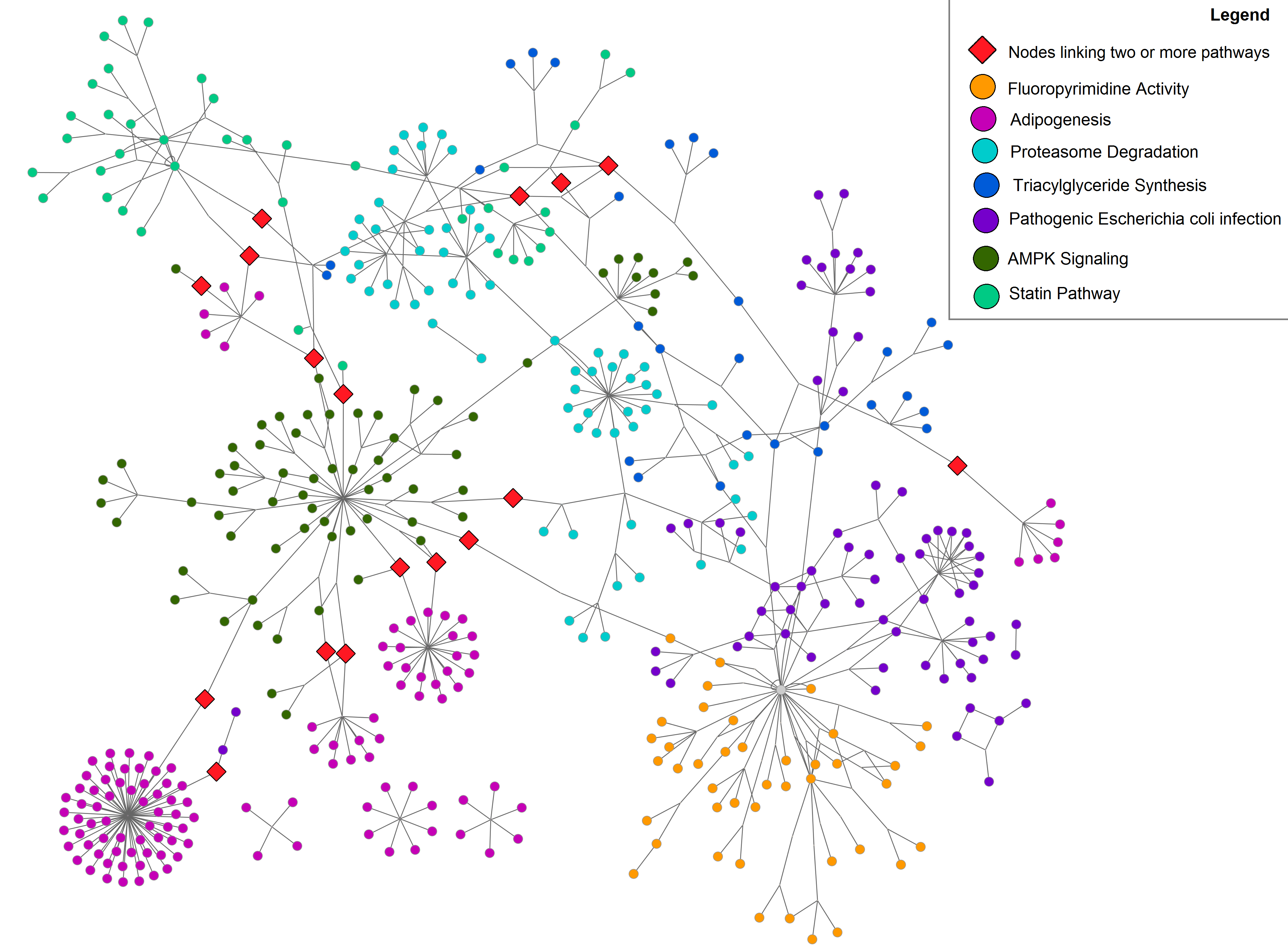

Supplement: Supplementary file 5 — Additional file 5: Figure S8. Integrated network of seven interconnected pathways that are changed in the diabetic fatty liver. (PNG 1 MB) [file 12864_2014_6667_MOESM5_ESM.png]
