# Supplementary material for: A network biology workflow to study transcriptomics data of the diabetic liver
Source: BMC Genomics. 2014 Nov 15;15(1):971. doi: 10.1186/1471-2164-15-971 (PMC4246458; doi:10.1186/1471-2164-15-971)
Supplement: Supplementary file 6 — Additional file 6: Table S3. List of genes present in two or more pathways. (PDF 35 KB) [file 12864_2014_6667_MOESM6_ESM.pdf]

Gene Name	Description	Function	log2FC	p-value	Link to NAFLD / T2DM (PubMed)
AGPAT2	1-acylglycerol-3-phosphate O-acyltransferase 2	An enzyme that plays an important role in the production of glycerophospholipids and triacylgycerols.	1.467	0.01	yes (24899308)
CDKN1A	Cyclin-dependent kinase inhibitor 1A	A potent cell cycle inhibitor and it plays a role in the induction and maintenance of cellular senescence.	1.226	0.01	yes (23142622)
SREBF1	Sterol Regulatory Element Binding TF 1	A transcription factor regulating genes required for glucose and fatty acids metabolism and lipid production.	1.027	0.03	yes (12855691)
LIPC	Hepatic lipase	An lipase expressed in the liver to convert IDL to LDL.	0.516	0.34	yes (18510853)
SLC2A4	Glucose transporter type 4	GLUT4 is an insulin-regulated glucose transport.	0.16	0.02	yes (23349036)
HMGCR	HMG-CoA Reductase	An enzyme controlling the cholesterol biosynthesis pathway.	0.107	0.8	yes (22560219)
LIPE	Hormone Sensitive Lipase	An enzyme responsible for the mobilization of stored lipids	0.14	0.03	yes (23688034)
TP53	Tumor Protein 53	A tumor suppressor protein regulating cell cycle arrest, apoptosis, senescence, DNA repair, or changes in metabolism.	0.085	0.27	yes (22641095)
ADIPOQ	Adiponectin	Adiponectin is regulating glucose levels as well as fatty acid breakdown.	-0.061	0.49	yes (23977033)
LEP	Leptin	Hormone which regulated the amount of fat stroed in the body.	-0.085	0.34	yes (24377452)
CTNNB1	Catenin	A protein regulating the coordination of cell-cell adhesion and gene transcription.	-0.085	0.73	yes (23433827)
LPL	Lipoprotein Lipase	An enzyme responsible for triglyceride hydrolase and a factor for lipoprotein uptake	-0.085	0.38	yes (19301078)
MEF2BNB-MEF2B	Myocyte Enhancer Factor 2B	The protein is thought to regulate gene expression, including expression of the smooth muscle myosin heavy chain gene.	NA	NA	NA
DGAT1	acyl CoA:diacylglycerol acyltransferase 1	A key enzyme in TG synthesis, in fatty liver development	NA	NA	yes (19472314)

Table S3: List of genes present in two or more pathways. Per gene the official gene symbol, a description of the gene and the function is given. Moreover the gene expression in the diabetic, fatty liver versus control is indicated as log2 fold change and the significance level as p-value. Finally, it is indicated whether the gene is known to be linked to NAFLD / T2DM.
