# Supplementary material for: A network biology workflow to study transcriptomics data of the diabetic liver
Source: BMC Genomics. 2014 Nov 15;15(1):971. doi: 10.1186/1471-2164-15-971 (PMC4246458; doi:10.1186/1471-2164-15-971)

Figure S9: Drug extension simulation for all drugs. A random network was created 10,000 times consisting of 408 genes that are present in at least one of the human pathways from WikiPathways. The network was then extended with drugs from Drugbank 4. This bar chart visualizes the frequency of how many drugs were added to the different random networks. The red line indicates the mean, the yellow line the standard deviation and the blue line represents the number of drugs added in the network that was used in the analysis of this paper. Since the random networks consist of genes present it at least one pathway, the number of drugs added to the network (blue) lies close to the mean of the distribution.

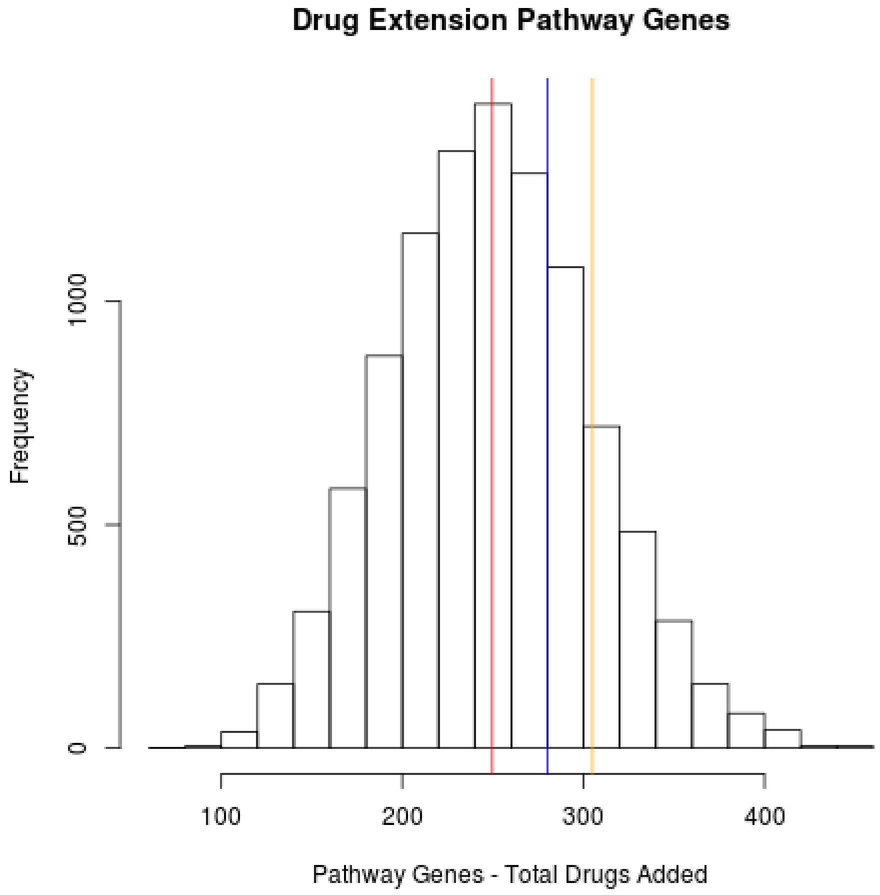

Figure S10: Drug extension simulation for antidiabetic drugs. A random network was created 10,000 times consisting of 408 genes that are present in at least one of the human pathways from WikiPathways. The network was then extended with *anti-diabetic* drugs from Drugbank 4. This bar chart visualizes the frequency of how many drugs were added to the different random networks. The red line indicates the mean, the yellow line the standard deviation and the blue line represents the number of drugs added in the network that was used in the analysis of this paper. The number of anti-diabetic drugs added to the network used in the analysis (blue) is significantly higher than random. The amount of 16 anti-diabetic drugs was not reached in any random network.

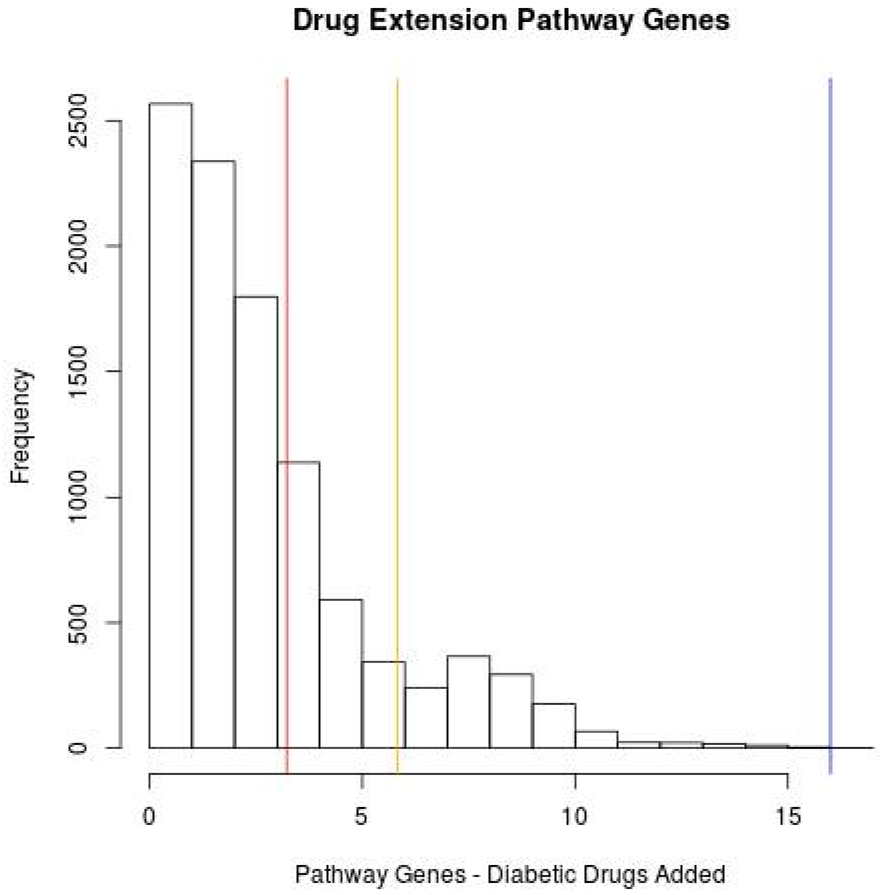

Supplement: Supplementary file 7 — Additional file 7: Figure S9. Drug extension simulation for all drugs. Figure S10. Drug extension simulation for antidiabetic drugs. (PDF 59 KB) [file 12864_2014_6667_MOESM7_ESM.pdf]
